# Contrasting roles of *GmNAC065* and *GmNAC085* in natural senescence, plant development, multiple stresses and cell death responses

**DOI:** 10.1038/s41598-021-90767-6

**Published:** 2021-05-27

**Authors:** Bruno Paes Melo, Isabela Tristan Lourenço-Tessutti, Otto Teixeira Fraga, Luanna Bezerra Pinheiro, Camila Barrozo de Jesus Lins, Carolina Vianna Morgante, Janice Almeida Engler, Pedro Augusto Braga Reis, Maria Fátima Grossi-de-Sá, Elizabeth Pacheco Batista Fontes

**Affiliations:** 1 Biochemistry and Molecular Biology Department, Universidade Federal de Viçosa, Viçosa, Brazil; 2 Embrapa Genetic Resources and Biotechnology, CENARGEN, Brasília, Brazil; 3 Pole Sophia Agrobiotech, Institute Nacional de la Recherche Agronomique, INRAE, Sophia Antipolis, France; 4 National Institute in Science and Technology in Plant-Pest Interactions, NCTIPP, Bioagro, Viçosa, Brazil; 5 Genomic Sciences and Biotechnology Program, Universidade Católica de Brasília, Brasília, Brazil; 6 National Institute in Science and Technology, INCT Plant Stress-Biotech, CENARGEN, Brasília, Brazil

**Keywords:** Biochemistry, Cell biology, Genetics, Molecular biology, Plant sciences

## Abstract

NACs are plant-specific transcription factors involved in controlling plant development, stress responses, and senescence. As senescence-associated genes (SAGs), NACs integrate age- and stress-dependent pathways that converge to programmed cell death (PCD). In Arabidopsis, NAC-SAGs belong to well-characterized regulatory networks, poorly understood in soybean. Here, we interrogated the soybean genome and provided a comprehensive analysis of senescence-associated *Glycine max* (Gm) NACs. To functionally examine GmNAC-SAGs, we selected *GmNAC065*, a putative ortholog of Arabidopsis *ANAC083/VNI2 SAG*, and the cell death-promoting *GmNAC085,* an *ANAC072* SAG putative ortholog, for analyses*.* Expression analysis of *GmNAC065* and *GmNAC085* in soybean demonstrated (i) these cell death-promoting *Gm*NACs display contrasting expression changes during age- and stress-induced senescence; (ii) they are co-expressed with functionally different gene sets involved in stress and PCD, and (iii) are differentially induced by PCD inducers. Furthermore, we demonstrated *GmNAC065* expression delays senescence in Arabidopsis, a phenotype associated with enhanced oxidative performance under multiple stresses, higher chlorophyll, carotenoid and sugar contents, and lower stress-induced PCD compared to wild-type. In contrast, *GmNAC085* accelerated stress-induced senescence, causing enhanced chlorophyll loss, ROS accumulation and cell death, decreased antioxidative system expression and activity. Accordingly, GmNAC065 and GmNAC085 targeted functionally contrasting sets of downstream *At*SAGs, further indicating that GmNAC85 and GmNAC065 regulators function inversely in developmental and environmental PCD.

## Introduction

Senescence is a programmed biological process characterized by cell, tissue and organ disassembly and degeneration, culminating in death^1, 2^. At late developmental stages or under stressful conditions, plant cells trigger an extensive genome reprogramming to initiate programmed cell death (PCD)^1, 3–6^. Recently, different sets of genes have been associated with age-triggered PCD, called developmentally induced PCD (dPCD), and environmentally triggered PCD (ePCD), a kind of PCD stimulated by environmental factors, including biotic or abiotic stresses, despite their common biochemical bases and phenotypes but different regulatory networks^5, 7^.

In plants, senescence is well understood in leaves, which display typical phenotypic changes with a consequent reduction in functionality until the abscission^2, 6^. The leaf senescence onset is accompanied by a dramatic reduction in metabolism and nutrient mobilization to growing and storage organs to support the reproductive phase^1^. In this process, chlorophyll degradation causes leaf yellowing and exposes carotenoids, including anthocyanins. Other typical symptoms are lipid peroxidation, high accumulation of reactive oxygen species (ROS), and high mobilization of soluble sugars, detected in stress- and age-induced senescence^8–10^. These events are precisely controlled by phytohormones, which integrate endogenous and exogenous signals throughout the activity of several senescence-promoting genes that control the onset, progression, and finalization of PCD^1, 2, 4, 11, 12^.

The control of plant stress responses and PCD is coordinated by several plant hormones. If the severity of the stress overcomes the efficiency of stress-avoiding and tolerance mechanisms, these signals, collectively, trigger premature senescence. The hormone signal transduction results in a global remodeling of gene expression profiles, affecting primarily the expression pattern of several TFs. The crosstalk among phytohormones, TFs, and other combinatorial factors imposes high complexity for understanding stresses-related pathways and molecular mechanisms governing plant senescence^1, 2^.

NAC and WRKY constitute the most expressive families of TFs in senescence, which include several characterized senescence-associated genes (SAGs)^1, 2, 13^. In *Arabidopsis thaliana*, more than 30 NAC genes display enhanced expression during dPCP^14^, and many of them are significantly up-regulated by at least one type of abiotic stress^14–17^. These analyses indicate that NACs can be critical regulatory TFs, which integrate developmental signals, stress responses, and PCD^1^.

The involvement of NAC TFs in senescence has also been reported in crops. In rice, *OsNAP* (Os03g21060) was associated with the onset of dPCD, drought, and biotic stress responses^18^. Overexpression of *OsNAP* causes precocious senescence and up-regulation of chlorophyll degradation- and JA metabolism-related genes^18–20^. Moreover, six rice NAC genes (*OsNAC005*, *OsNAC006*, *OsNAC009*, *OsNAC010*, *OsNAC011*, and *ONAC106*) are involved in both abiotic stress response and senescence^21–24^. In soybean, *GmNAC030* and *GmNAC081* are involved in both dPCD and ePCD and are induced by ER stress and osmotic stress^25, 26^. These NAC TFs belong to a developmental cell death (DCD) domain-containing N-rich protein (NRP)-mediated circuit, with culminates in VPE (vacuolar processing enzyme) enhanced expression^25, 27–29^. VPE is a caspase-like 1 protein that executes a plant-specific PCD throughout the vacuole collapse^26, 30, 31^.

Global gene expression analysis of senescing soybean leaves demonstrated that 40% of NAC genes, including *GmNAC030* and *GmNAC081*, are differentially expressed during leaf senescence^32, 33^. Other uncharacterized NAC genes display a strong relationship with multiple stress responses and cell death progression. *GmNAC065* and *GmNAC085*, which are putative orthologs of the SAGs *ANAC083/VIN2* and *ANAC072*, respectively, display contrasting patterns of expression under different stresses and in different tissues in soybean plants^33^. The contrasting expression profiles of *GmNAC065* and *GmNAC085* soybean genes are also observed in seedlings subjected to different stresses^33^. When transiently expressed in *Nicotiana benthamiana* leaves, they trigger typical symptoms of senescence, including leaf yellowing, chlorophyll loss, ROS production, and lipid peroxidation. Furthermore, *GmNAC065* and *GmNAC085* are phylogenetically related to negative and positive regulators of senescence in Arabidopsis, respectively. These opposing functional and expression profiles make them valuable targets for functional studies of senescence mechanisms and biotechnological breeding in soybean.

Despite the involvement of NAC TFs in multiple stress responses, dPCD and ePCD in crops, several regulatory mechanisms of PCD in plants are still unclear. The soybean genome encompasses more than 180 NAC members^33^, which have been often associated with multilayered signaling events in stressful conditions, making them functionally complicated, poorly understood, and frequently unfeasible as a target for biotechnological intervention. Here, we identified putative NAC SAGs in soybean and validated their association with PCD by transcriptome-wide analyses in different environmental conditions. Furthermore, we functionally characterized two GmNAC SAGs, *GmNAC065* and *GmNAC085*, in plant development, multiple stress responses, and cell death progression.

Our data demonstrate that *GmNAC065* and *GmNAC085* are induced by different stress conditions, with different kinetics and extent of responses, playing contrasting roles in PCD. They displayed a divergent expression profile from other SAGs and downstream target genes implicated in the antioxidant system and photosynthetic apparatus. Collectively, our data provided new insights into the soybean NAC-mediated PCD and reinforced the importance of these TFs as targets for understanding PCD regulatory gene networks and biotechnological plant breeding.

## Material and methods

### Identification and phylogenetic analysis of NAC-SAGs in soybean

The identification and the phylogenetic analysis of NAC-SAGs in soybean were performed using *Arabidopsis thaliana* (*At*)SAGs as templates. The deduced amino acid sequences of previously described AtNAC-SAGs^2^ were accessed from TAIR database (https://www.arabidopsis.org/) and used as query sequences against the soybean genome. For a global alignment analysis, we used Phytozome’s BLASTp algorithm (https://phytozome.jgi.doe.gov/pz/portal.html#!search?show=BLAST&method=Org_Gmax). The putative recovered GmSAGs were confirmed by the best score of sequence-similarity (e-value < 10^–10^) and alignment cover-ratio (coverture > 90%). Using the Soybase dataset (https://soybase.org/), we identified the putative GmNAC-SAGs paralogous genes and, finally, AtNAC-SAGs and GmNAC-SAGs were phylogenetically analyzed. The phylogenetic analysis and tree rendering were conducted using the MABL online phylogeny-platform (http://www.phylogeny.fr/simple_phylogeny.cgi?workflow_id=e72d406522a15231c99e208ac9396b27&tab_index=6&go_next=1#anchor) supported by the MUSCLE alignment algorithm and the neighbor-joining statistical method with 10,000 bootstraps. We also used *GmNAC081* and *GmNAC030* as unrelated AtNAC-SAGs but as age- and stress-induced senescence-associated genes in soybean^26, 32^. The numerical designation of the superfamily GmNAC members was defined by Le et al.^34^ and further complemented by Melo et al.^33^.

### Transcriptome-wide analysis of soybean NAC-SAGs in response to multiple stresses

The expression levels of the putative GmNAC-SAGs were retrieved from eight differentially expressed gene (DEG) soybean datasets available in public transcriptome raw-data from GEO—Gene Expression Omnibus/NCBI (https://www.ncbi.nlm.nih.gov/geo/), comprising different types of abiotic and biotic stresses, age- and hormone-induced senescence. DEGs were determined using the edgeR package for gene expression analysis, and differential gene expression was supported by F and t-tests. Target genes were only searched against annotated DEGs after FDR (false discovery rate) correction and considered differentially expressed with 1 < FC < − 1 and p-value < 0.05. All stress conditions, soybean treatments, access numbers, and references used in the transcriptome-wide analysis are organized in Supplementary Table 1.

For *GmNAC065* and *GmNAC085*, we performed a co-expression analysis of soybean plants submitted to different abiotic and biotic stresses using the EXPath 2.0 platform. We used Pearson's correlation-method with a cut-off of 0.8 (positive/negative) to determine the positive and negative correlated genes. The correlated genes were organized by biological function and process according to the internal GO enrichment analysis.

### Soybean growth and multiple stress assay

Soybean (*Glycine max*—Williams82) seeds were soil-germinated and grown under greenhouse conditions (12 h of light, 15–30 °C, 70% relative humidity). At the V2/V3 developmental stage, the roots were acclimated in Hoagland Hydroponic Solution (HHS) for 24 h prior to stress simulations. The stress assay was performed in a hydroponic system, with HHS supplemented with 10% (w/v) PEG (MW 8000) to induce osmotic stress, 5 μg/mL tunicamycin (Tun) to induce ER-stress, the phytohormones ABA (150 mM) and salicylic acid (SA—75 µM), to trigger drought and biotic stress-responses, respectively. For the air-dry treatment, the plants were removed from the HHS and placed on cotton-filled pots. Leaf discs of stressed and control (0 h—untreated plants) seedlings were collected after 2 h, 4 h, and 12 h of stress-treatment, immediately frozen in liquid nitrogen, and stored at − 80 °C. All treatments were performed with biological triplicates, consisting of three pools of three plants. For induced PCD assay, the soybean seedlings in the same growth conditions were subjected to bleomycin treatment (420 mM) in HSS.

### Soybean RNA-extraction, cDNA synthesis, and gene expression analysis

Total RNA of soybean leaf disks was extracted from approximately 150 mg of tissue, according to the Trizol^®^ (Invitrogen) manufacturer’s recommendations. RNA quality access and cDNA synthesis were performed as previously described by Melo et al.^33^.

The gene expression profiles were determined by qRT-PCR. The analyses were performed in QuantStudio 3 instrument (ThermoFischer), with GoTaq qPCR master mix (Promega) and the specific primers (Supplementary Table 2). For experiment accuracy, three independent cDNA pools for each treatment were used. *ELF1A* soybean gene was used as the endogenous control, with stability determined by Freitas et al.^35^. Relative gene expression was determined by the 2^−ΔΔCt^ or 2^−ΔCt^ method and gene expression results converted into a heatmap using MORPHEUS software (https://software.broadinstitute.org/morpheus/).

### Arabidopsis GmNAC-overexpressing lines

*Arabidopsis thaliana* (ecotype Columbia) transgenic lines ectopically expressing the genes *GmNAC065* and *GmNAC085* (designated GmNAC065-OX and GmNAC085-OX, respectively) were generated by the floral dip method^36^. The previously obtained recombinant plasmids^33^ were used to transform *Agrobacterium tumefaciens* (GV3101 strain), selected on LB-agar medium supplemented with specific antibiotics, and used for a three-round transformation process, with an interval of 10 days between the rounds. Transgenic plants with a T-DNA insertion were selected by ammonium-glufosinate pulverization (120 mg/L) and confirmed by PCR. Three T_2_ homozygous independently transformed lines were obtained for each construction, and the gene expression level was monitored by qRT-PCR.

### GmNAC065-OX and GmNAC085-OX phenotypic characterization

For the phenotypic characterization, the seeds of GmNAC065-OX, GmNAC085-OX, and Col 0, previously refrigerated at dark for dormancy-broken, were soil-sowed and germinated under a controlled growth chamber (12 h of photoperiod, 21 °C and 70% of relative humidity). After germination, two-week-old seedlings were carefully transferred to individual pots, randomly distributed in trays, and cultivated up to 70 days (10 weeks). During the developmental cycle, we analyzed: (i) rosette diameter; (ii) the number of leaves; (iii) shoot length; (iv) duration of the vegetative stage; (v) duration of the reproductive stage; (vi) senescence onset. These parameters were monitored according to natural plant life-stages' progression in intervals of 7 days between measurements.

### Arabidopsis stress treatment, RNA extraction, and cDNA synthesis

Four-week-old seedlings were organized in three pools of three plants for each treatment. Plant germination and growth were performed according to the phenotypic and morphological analyses section. For the stress assay, the seedlings were carefully removed from the soil and transferred to MS medium (2.3 g/L of MS basal medium) for 24 h for plant acclimation. After the acclimation, the plants were transferred to PEG, Tun, or SA solutions and collected after 24 h. Total RNA extraction and cDNA synthesis were performed as described in “Soybean growth and multiple stress assay” section.

### Biochemical and cellular analysis of plant antioxidative system

The plant antioxidative system was evaluated by different approaches, including H_2_O_2_-DAB leaf-staining, TBA-reactive compounds quantification, enzymatic activity analysis of superoxide dismutase (SOD), catalase (CAT), and ascorbate peroxidase (APX). For H_2_O_2_ detection, the plants were soaked in 3,3-diaminobenzamidine (DAB) solution (1 mg/mL, pH 3.8) for 8 h under continuous agitation. After staining, the leaves were immersed into a 100% (v/v) ethanol bath until total chlorophyll removal. The leaves were rehydrated in water and glycerol (10% v/v) and photographed in a stereoscope.

TBA-reactive compounds from approximately 100 mg of tissue were quantified as optimized by Melo et al.^33^. The concentration of TBA-reactive compounds was calculated considering the MDA molar extinction coefficient of 155 mM cm^−1^.

The enzymatic activities were determined in the total protein extract, as described by de Melo et al.^37^. The proteins from approximately 150 mg of leaves were extracted in 1 mL of phosphate buffer (100 mM—pH 7.8) and added to DTT (0.5 g/L), EDTA (10 µM) and PVPP (0.3 mg/mL). All enzymatic activities were determined using pools of 3 plants and three biological and two technical replicates. Total protein quantification was performed by Bradford’s method.

From data of total protein quantification, we determined the rate of protein degradation. The following expression was applied: (%) of protein degradation = $\frac{100 \cdot (P_{0}-P_{n})}{P_{0}}$, P_0_ = total protein of unstressed plants and P_n_ = total protein at specific stress-time.

### Cellular analysis of cell death progression

Cell death progression was monitored in leaves and roots by Evans blue and propidium iodide (PI) staining. The Evans blue leaf-staining was performed in Evans dye aqueous solution (0.5% w/v). Stressed (24 h) and non-stressed leaves were immersed into the staining solution for 8 h under agitation. After staining, the leaves were distained in absolute ethanol and preserved in glycerol (50% v/v) until observation under a stereoscope.

For the PI root-staining, 24 h-stressed plants were dipped into propidium iodide solution (0.6 µg/mL) for 24 h. After staining, the plants were rinsed in distilled water, and roots were arranged at slides under a water drop. The slides were covered and analyzed by confocal microscopy scanning (Zeiss LSM510 META) under argon/helium–neon laser with a maximum excitation wavelength of 488 nm. Fluorescence was collected in a 596–638 bandpass filter. Images were recovered and treated using the ZEN BLACK EDITION 3.0 (Carl-Zeiss) software.

### Secondary metabolites quantification

Metabolite content was monitored in ethanolic plant-extracts. The levels of chlorophyll, carotenoids, soluble sugars and anthocyanins were determined using the same leaf extract. The frozen samples were weighted, powdered in liquid nitrogen, and metabolites extracted in ethanol for 24 h under refrigeration and sheltered from the light.

Chlorophyll, carotenoids, and anthocyanin levels were determined spectrophotometrically. The supernatant was quantified using a spectrophotometer at 480 nm, 645 nm, and 663 nm. Chlorophyll a, b, and total chlorophyll were calculated by the expression *Clf*_*a*_ = $\frac{(12.7 \cdot A663)-(2.69 \cdot A645)}{m}$; *Clf*_*b*_ = $\frac{(22.9 \cdot A645)-(4.68 \cdot A663)}{m}$; *Clf*_(*a*+*b*) =_ $\frac{(20.2 \cdot A645)+(8.02 \cdot A663)}{m}$, respectively, and expressed as µg/mg of tissue. To calculate the concentration of carotenoids, we used the equation *Car* = $\frac{[10\cdot A480]}{\varepsilon \cdot m}$, considering ε = 2592 mM cm^−1^ for standard carotenoids in ethanol. Finally, anthocyanin content was determined according to the method adapted by Sims and Gamon^38^. The samples were analyzed at 529 nm and 650 nm and the final concentration of anthocyanins calculated by the expression *Cyan* = $\frac{A529-0.288 \cdot A650}{\varepsilon \cdot m}$, in which m = tissue weight (mg) and ε = 98.2 mM cm^−1^ (molar extinction coefficient determined for a mixture of standard-anthocyanins in acidic solution.

For reducing sugar quantification, we performed a DNS-based (3,5-dinitrosalycilic acid) reaction, as described by de Melo et al.^37^. An aliquot of 100 µL of the extract was added to 900 µL of water and 500 µL of DNS. Absorbance was determined at 540 nm, and sugar content was determined using a glucose-based standard curve (0–1.2 µg/µL).

### Quantitative RT-PCR

The expression of SAGs and downstream targets in Arabidopsis was determined as previously described in “Soybean RNA-extraction, cDNA synthesis, and gene expression analysis” section using gene-specific primers (Supplementary Table 3). *ACT2* was selected as the normalizer control gene. The expression of the drought-responsive genes *RD29A*, *RD29B*, and *RD20*, ER stress-induced *ANAC036*, *CNX*, and *AtNRP1* genes, and biotic stress-associated marker genes *WRKY70*, *AtNRP1*, and *RAB18* were monitored by RT-qPCR.

### Ethical statement

This work adhered all the protocols relevant to ethical guidelines/regulations for plant usage.

## Results

### Genome- and transcriptome-wide analysis identified soybean NAC genes associated with developmental and environmental senescence

In Arabidopsis, several studies of temporal gene expression profiling along with dPCD and ePCD have associated NAC TFs with the control of the onset, progression, and termination of PCD^1, 2, 5^. In order to identify the soybean NAC TFs associated with the control of PCD in developmental programs and multiple stresses responses, we performed a genome and transcriptome-wide analysis of soybean under different stresses. Kim et al.^1^ and Bengoa-Luoni et al*.*^2^ provided a complete inventory of senescence-associated NAC genes (NAC-SAGs) in *Arabidopsis thaliana*. Here, we used these functionally characterized *At*NAC-SAGs as templates, and we identified 16 putative *Gm*NAC-SAGs in the soybean genome (Table 1).

**Table 1:** Senescence-associated genes in *Arabidopsis thaliana* and soybean (*Glycine max*).

Group	NAC-SAG (*Arabidopsis thaliana*)	NAC subgroup*	Soybean putative NAC-SAG	Locus	Soybean paralogous gene	Locus
1	*ANAC016*	NAM	*GmNAC074*	Glyma.10G219600	*GmNAC149*	Glyma.20G172100
2	*ANAC059/ORS1*	NAM	*GmNAC023*	Glyma.05G025500	**	–
	*ANAC092/ORE1*	NAM	*GmNAC131*	Glyma.17G101500	**	–
3	*ANAC042/JUB1*	ONAC022	*GmNAC106*	Glyma.14G030700	*GmNAC154*	Glyma.02G284300
4	*ANAC083/VNI2*	Senu5	*GmNAC065*	Glyma.08G360200	*GmNAC179*	Glyma.18G301500
5	*AtNAP*	SNAC-B	*GmNAC010*	Glyma.02G109800	*GmNAC003*	Glyma.01G051300
6	*ATAF1*	SNAC-A	*GmNAC109*	Glyma.14G152700	*GmNAC011*	Glyma.13G030900
7	*ANAC072*	SNAC-A	*GmNAC085*	Glyma.12G149100	*GmNAC043*	Glyma.06G248900
	*ANAC019*	SNAC-A	*GmNAC101*	Glyma.13G279900	***	–
	*ANAC055*	SNAC-A	*GmNAC092*	Glyma.12G221500	***	–

*According to Melo et al. (2018).

**GmNAC023 and *GmNAC131* are putative soybean paralogous genes.

***GmNAC092 and *GmNAC101* are putative soybean paralogous genes.

**Figure 1:**
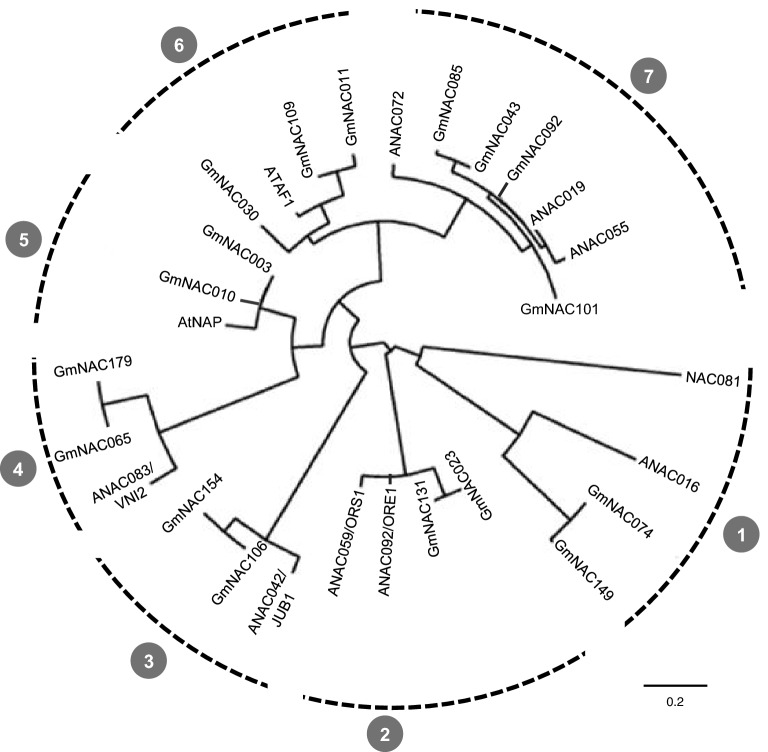
Phylogenetic reconstruction of *Arabidopsis thaliana* and soybean (*Glycine max*) NAC-SAGs. The deduced amino acid sequences of previously described NAC-SAGs of Arabidopsis were used as prototypes to retrieve their putative orthologous genes (*Gm*NAC-SAGs) from the soybean genome. The phylogenetic reconstruction resulted in seven distinct groups with stable collapsed branches. Phylogenetic relationships were established by the neighbor-joining statistical method with 10,000 bootstraps, and the tree was rendered using the MABL interface.

The last phylogenetic analysis of the soybean NAC superfamily updated the family to 180 members, divided into 15 subfamilies, according to sequence and functional relationship^33^. The phylogenetic analysis of GmNAC-SAGs clustered the putative genes in 7 phylogenetic groups, whose members belong to 5 different NAC subfamilies (Fig. 1). The most representative senescence-associated NAC subfamily is SNAC-A, followed by SNAC-B subfamilies. SNAC-A and SNAC-B subfamilies contain 50% of putative GmNAC-SAGs, divided into three closely related groups (5, 6, and 7—Fig. 1). *GmNAC010* and *GmNAC003* belong to the SNAC-B subfamily, whereas *GmNAC109*, *GmNAC011*, *GmNAC085*, *GmNAC043*, *GmNAC101*, and *GmNAC092* are SNAC-A subfamily members. These clusters also harbor the Arabidopsis genes *AtNAP* (group 5), *ATAF1* (group 6) and *ANAC072*, *ANAC019*, and *ANAC055* (group 7), functionally associated with senescence progression and several stress responses^39–42^.

Subsequently, the NAM subfamily holds 25% of NAC-SAGs divided into groups 1 and 2 (Fig. 1). The NAM subfamily encompasses the genes *GmNAC074, GmNAC023, GmNAC131*, and *GmNAC149*, besides their orthologous in Arabidopsis *ANAC016*, *ANAC059/ORS1* and *ANAC092/ORE1*, respectively. Finally, groups 3 and 4 belong to two different NAC subfamilies. *GmNAC106* and *GmNAC154* (group 3—Fig. 1) are phylogenetically related to ONAC022 subfamily, and *GmNAC065* and *GmNAC179* (group 4—Fig. 1) belong to the Senu5 subfamily.

The analysis of putative GmNAC-SAGs in soybean genome did not identified *GmNAC030* and *GmNAC081*, the final regulators of the NAC-VPE circuit as classical NAC-SAGs. *GmNAC081* belongs to the TERN subfamily and is clustered with a most divergent branch of the phylogenetic tree (group 1—Fig. 1). However, *GmNAC030* belongs to the SNAC-A subfamily, covered by the members of the groups 6 and 7, but it displays some structural divergence that promotes a branch collapse and places it in a new phylogenetic branch.

**Figure 2:**
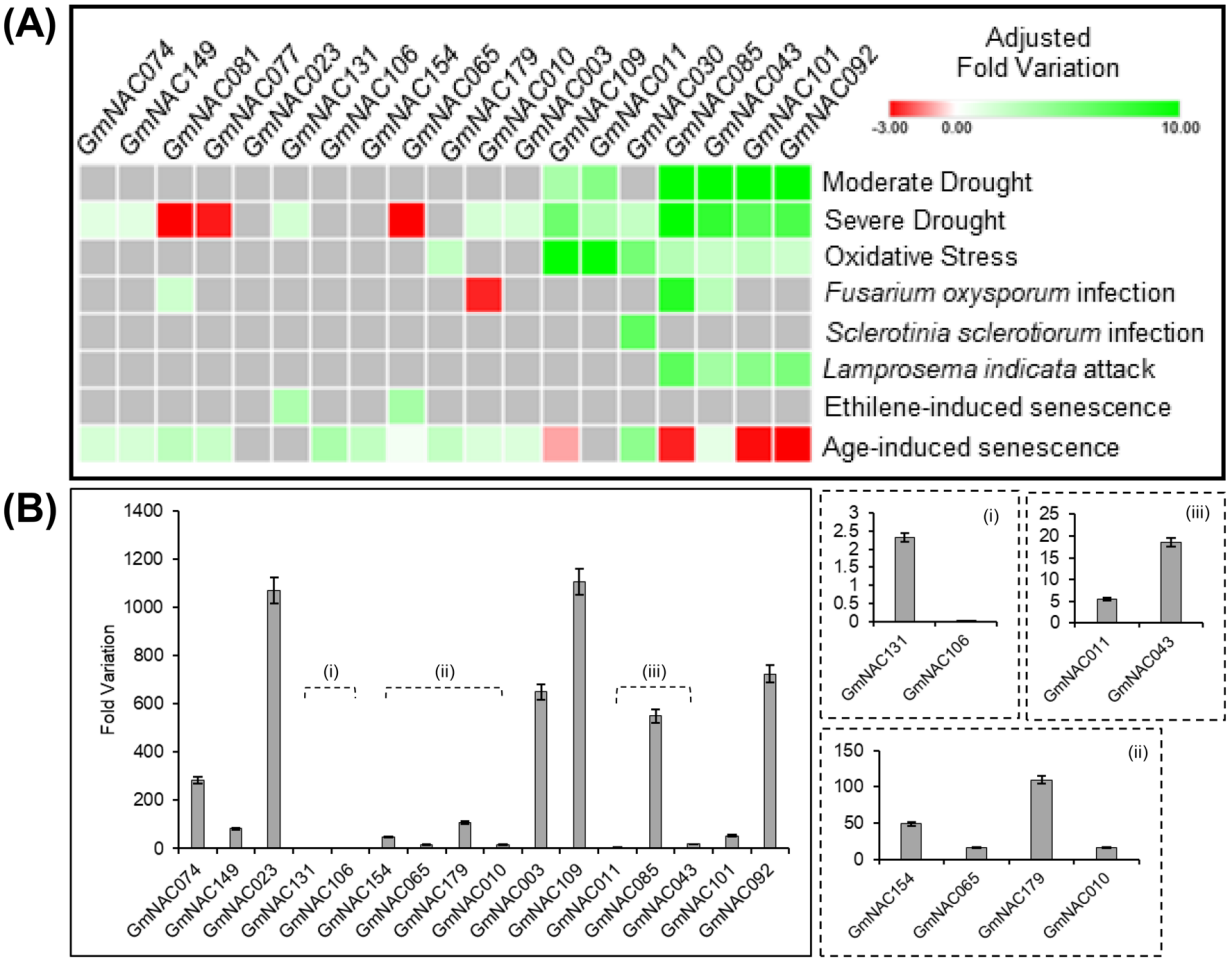
Expression pattern of GmNAC-SAGs under different stresses and bleomycin-induced cell death in soybean. (**A**) Heatmap of GmNAC-SAGs expression in soybean exposed to different stresses. Transcriptome-wide data from publically accessed RNAseq repository on NCBI were accessed, and the expression of *Gm*NAC-SAGs investigated under drought (moderate and severe), oxidative stress, biotic stresses (fungi and insect attack), and senescence (ethylene-induced and age-induced) conditions. Fold variation values from DEGs were recovered and converted into the heatmap. (**B**) Expression profile of GmNAC-SAGs in soybean seedlings exposed to bleomycin-treatment (420 mM) for 24 h.

To identify NAC TFs associated with the regulatory mechanisms of dPCD and ePCD in soybean, we analyzed the expression profile of the putative GmNAC-SAGs from a publically accessed soybean-transcriptome dataset from the GEO (NCBI) database (Supplementary Table 1, Fig. 2). Their expression profiles were assessed in response to different stress conditions and natural senescence (dPCD), such as hormone-induced senescence, abiotic stresses (moderate and severe drought conditions, oxidative stress), and biotic stresses (fungi and insect attacks). In addition, their expression levels were also examined in soybean seedlings treated with bleomycin, a genotoxic PCD-inducer.

Our transcriptome analysis demonstrated that GmNAC-SAGs are differentially expressed at least in one type of abiotic and/or biotic stresses, and most of them are differentially expressed during age-induced senescence (Fig. 2A). We also monitored the expression of *GmNAC081* and *GmNAC030* as marker genes of stress- and leaf senescence-induced genes^43^. *GmNAC074*, *GmNAC149*, *GmNAC010*, and *GmNAC003* display similar expression patterns in response to different stresses and during leaf senescence, which partially overlap with the GmNAC30 expression profile (Fig. 2A). In general, the selected GmNACs were differently regulated by age-induced senescence and, in response to the stress conditions, they exhibited a pattern of expression consistent with their subfamily. The SNAC-A genes within groups 6 and 7 were up-regulated during drought and oxidative stresses. Specifically, *GmNAC085*, *GmNAC043*, *GmNAC101*, and *GmNAC092* also displayed a similar expression pattern during biotic stresses (*Fusarium oxysporum* and *Lamprosema indicata* infection, Fig. 2A). In contrast, these genes were down-regulated during age-induced senescence and we have previously shown that they are also down-regulated by natural leaf senescence^33^; thereby, they were not conceptually classified as SAGs. An opposite trend was observed for *GmNAC065*, *GmNAC179*, *GmNAC081*, and *GmNAC077* (groups 1 and 4, respectively, Senu5 and TERN)*.* These genes were down-regulated by severe drought and up-regulated during age-induced senescence (this work) and natural leaf senescence^33^, hence we referred to them as GmNAC-SAGs (Fig. 2A). Because GmNAC065 and GmNAC085 have been previously demonstrated to induce cell death in a heterologous plant system^33^ and yet display a contrasting expression profile under natural and drought-induced senescence^33, 43^, we selected these cell death-related GmNACs for further analyses.

### Bleomycin-induced cell death differentially regulates GmNAC065 and GmNAC085

We hypothesized that the differentially expressed GmNACs in age-induced and natural leaf senescence were differently involved in dPCD and ePCD. To examine this hypothesis, we analyzed first their expression levels in soybean leaves treated with the cell death inducer bleomycin for 24 h (Fig. 2B). All examined GmNACs were induced by bleomycin treatment, although to a different extent. The only exception was *GmNAC106*, which was negatively regulated by the treatment. Some genes, including *GmNAC023* (group 2—NAM) and *GmNAC109* (group 6—SNAC-A), were strongly induced (more than 1000-fold variation), indicating involvement of these genes with the process of stress-induced PCD. *GmNAC003* (group 5—SNAC-B), *GmNAC085*, and *GmNAC092* (group 7—SNAC-A) were also induced by bleomycin treatment, as expected for the members of S-NAC subfamily already described as positive regulators of ePCD, such as *ANAC016*, *ORE1*, *ORS1*, and *ATAF1*^2, 40, 41, 44^. In contrast, the genes *GmNAC106* and *GmNAC154*, which clustered together with the Arabidopsis negative regulator of senescence JUB1 (ONAC022)^45^, displayed low induction compared with the other GmNACs-genes; likewise, *GmNAC065* and *GmNAC179* (group 4—Senu5), which clustered with another negative regulator of senescence in Arabidopsis (*ANAC083/VNI2*)^46^, were slightly induced by bleomycin.

### GmNAC065 and GmNAC085 are differentially expressed in soybean tissues during normal growth and stressful conditions

**Figure 3:**
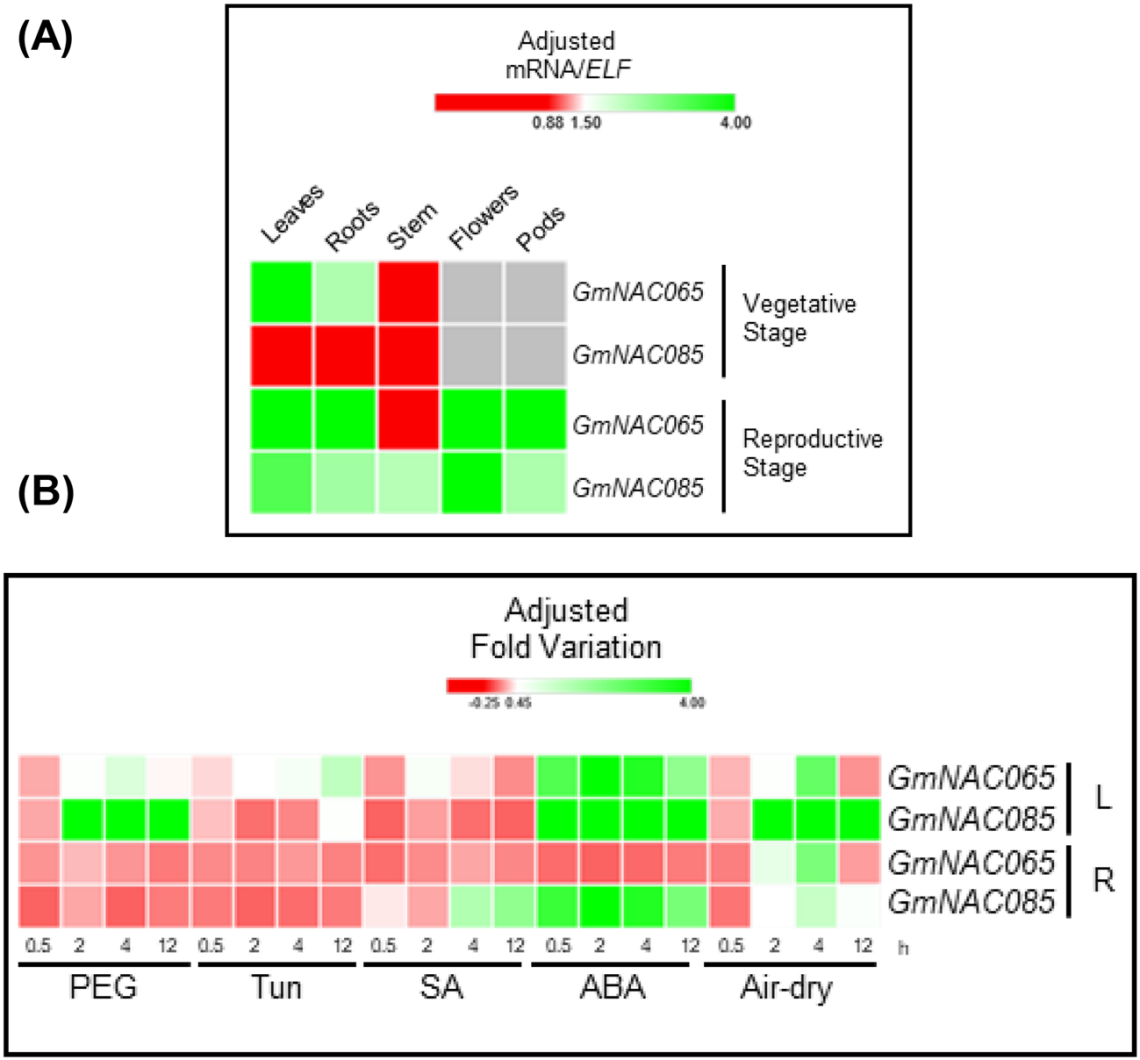
Tissue-specific expression of *GmNAC065* and *GmNAC085* in Williams 82 soybean seedlings under normal developmental conditions and different stresses. (**A**) Expression pattern of *GmNAC065* and *GmNAC085* in soybean vegetative and reproductive stages. Gene expression was determined by RT-qPCR (data available in Supplementary Figure 2A) and calculated using 2^−ΔCt^ method and *ELF1A* as the endogenous normalizer gene. (**B**) Expression pattern of *GmNAC065* and *GmNAC085* in soybean seedlings subjected to simulated drought (PEG 8000—10% w/v; air-dry and ABA—150 mM), ER stress (Tun—5 µg/mL), and biotic stress (SA—75 µM). The gene expression level was analyzed after 2 h, 4 h and 12 h treatments in leaves and roots by RT-qPCR (data available in Supplementary Figure 2B). Fold variation was calculated using the 2^−ΔΔCt^ method in three biological replicates and two technical replicates and normalized by Z-score considering gene expression in untreated plants.

To examine further the differential involvement of GmNACs in ePCD and dPCD, we selected *GmNAC065* and *GmNAC085*, which exhibited opposing expression profiles in response to stress and natural senescence^33^, for further analysis (Fig. 2A). We investigated their expression profile in different tissues of the Williams82 soybean-variety under normal and stressful conditions (Fig. 3, Supplementary Figure 1A). *GmNAC065* is expressed in all tissues during the vegetative and reproductive stages (Fig. 3A). The only exception is the stem, in which its expression is barely detected. In the vegetative stage, the expression of *GmNAC065* is higher in leaves than in roots. However, in the reproductive stage, the expression of *GmNAC065* is similar amongst all tissues, displaying a discrete variation. In contrast, *GmNAC085* displays a very low expression in leaves and roots during the vegetative stage, but a significantly enhanced expression in all tissues during the reproductive stage (Fig. 3A). Like *GmNAC065*, the expression of *GmNAC085* is higher in leaves than in roots in the reproductive stage, indicating a possible involvement in the stress-triggered senescence process, which appears to be more extensive in leaf tissue.

We also determined the expression levels of *GmNAC065* and *GmNAC085* in soybean seedlings exposed to PEG, air-dry, and ABA-treatments, simulating drought. ER-stress was induced by tunicamycin treatment, and biotic stresses were simulated by salicylic acid treatment. Our analyses were performed in four time-points (0.5 h, 2 h, 4 h, and 12 h) to determine whether these genes were early, intermediate or late stress-responsive.

GmNAC065 and GmNAC085 are regulated by at least one type of stress treatment and respond with different levels and kinetics of induction in leaves and roots (Fig. 3B, Supplementary Figure 1B). Both genes are most expressed in leaves than in roots, as observed under normal growth conditions. In leaves, *GmNAC065* was weakly up-regulated by PEG, Tun, and air-dry treatments. GmNAC065 displays an expressive induction during the early-to-intermediate ABA treatment stages but decays at a late-stage post-treatment. During the PEG treatment, *GmNAC065* responded with early-to-intermediate induction kinetics, displaying the highest expressions between 2 and 4 h, similar to air-dried and ABA-treated plants. Differently, in ER-stress, *GmNAC065* was only induced in late stages of stress, after 12 h of treatment. In contrast, *GmNAC085* was strongly induced by PEG, ABA, and dry air treatments, but not by Tun and SA in leaves. The temporal expression pattern was also different; GmNAC085 displays an intermediate-to-late response, with higher expression levels between 4 and 12 h, when *GmNAC065* expression decays (Fig. 3B). In roots, these NAC genes are weakly expressed. Nonetheless, *GmNAC085* responds to SA treatment at late stages and displays a stable expression during ABA-treatment.

### Co-expression analyses uncover different set of genes associated with *GmNAC065* and *GmNAC085* expression

We next performed a co-expression analysis in publically accessed RNA-seq and Microarray data of soybean under biotic and abiotic stresses. Differentially expressed genes (DEGs) were subjected to Pearson’s correlation method, and positively and negatively correlated genes were grouped according to their biological function and process by GO enrichment analysis (Table 2).

**Table 2:** *GmNAC065* and *GmNAC085* co-expressed genes in soybean under biotic and abiotic stresses.

GO ID	GO term	Gene	Functional annotation	P-value*
**GmNAC065—positive correlation (PCC ≥ 0.8)**				
0004871	Signal transducer	Glyma.15G074900	Protein kinase superfamily	5.93E^−3^
		Glyma.12G187600	LEA hydroxy-proline rich glycoprotein	
0055114	Oxidative/reductive process	Glyma.16G179100	β-Carotene-hydroxylase 1	0.05
		Glyma.01G025500	2-Oxoglutarate and Fe(II)-dependent oxygenase	
		Glyma.04G123800	Ubiquinol oxidase	
		Glyma.06G285400	Acyl-CoA oxidase 3	
**GmNAC065—negative correlation (PCC ≤ − 0.08)**				
0046423	Oxidative/reductive process	Glyma.01G086900	Allene-oxide cyclase 3	1.18E^−5^
0009740	GA-mediated signaling pathway	Glyma.10G241100	Gibberillin-oxidase 2 (A44)	2.96E^−4^
		Glyma.13G371400	GA_3_-recquiring oxidase	
**GmNAC085—positive correlation (PCC ≥ 0.8)**				
0009808	Lignin metabolic process	Glyma.02G236500	Trans-cinnamate 4-monooxygenase	2.13E^−4^
0009073	Aromatic amino acid biosynthesis	Glyma.11G189100	Prephenate dehydratase	4.48E^−4^
		Glyma.14G176600	3-Deoxy-7-phosphoheptulonate synthase	
		Glyma.05G183700	Shikimatekinase	
0009737	ABA-responsive genes	Glyma.03G056000	EM-likeprotein GEA6	1.66E^−3^
		Glyma.04G009900	Dehydrin	
		Glyma.18G203500	EM-likeprotein GEA1	
000502	Proteasome complex	Glyma.06G171200	20S proteasomesubunit beta 5	1.88E^−3^
		Glyma.11G227700	26S proteasome regulatory subunit N8	
		Glyma.01G124900	20S proteasomesubunit alpha 3	
		Glyma.06G078500	20S proteasomesubunit beta 1	
		Glyma.17G252100	20S proteasomesubunit alpha 6	
0004175	Endopeptidase activity	Glyma.12G072600	Sentrin-specific protease 8	1.99E^−3^
0042744	Hydrogen peroxide catabolic process	Glyma.U021900	L-ascorbate peroxidase	2.62E^−3^
		Glyma.06G275900	Peroxidase	
0005741	External mitochondrial membrane	Glyma.19G020100	Voltage-dependent anion-selective channel	3.56E^−3^
		Glyma.05G241100	Mitochondrialimport receptor subunit TOM40	
0043248	Proteasome assembly	Glyma.16G126300	26S proteasome regulatory subunit N10	0.02
		Glyma.09G210000	26S proteasome regulatory subunit N9	
0051603	Proteolysis involved in cellular protein catabolic process	Glyma.04G155900	Serine-carboxypeptidase-like 29	0.08
		Glyma10G207300	Serine-carboxypeptidase-like 47	
**GmNAC085—negative correlation (PCC ≤ − 0.08)**				
0016117	Carotenoid biosynthetic process	Glyma.03G128900	Lycopene β-cyclase	2.60E^−4^
		Glyma.02G188200	Prolycopene isomerase	
0009507	Chloroplast	Glyma.11G195900	Chaperonin 60 subunit alpha 1	2.14E^−3^
		Glyma.06G159400	GTP-binding protein LEPA	
		Glyma.06G039700	Photosystem I light-harvesting complex 5	

*P-value of statistical analysis of FDR (false discovery rate) for GO-enrichment.

*GmNAC065* is positively correlated with genes related to primary cell signaling pathways, such as kinase proteins, and genes associated with cell survival in multiple stresses tolerance. The analysis indicates its co-expression with LEA proteins, β-carotene hydroxylase, acyl-CoA oxidase, and ubiquinol oxidase. These proteins and enzymes are frequently associated with ROS-avoiding mechanisms, photosynthetic apparatus preservation, jasmonate-mediated tolerance to biotic stresses, and drought-tolerance^47–49^. *GmNAC065* negatively correlates with one gene of jasmonic acid biosynthesis (allene-oxide 3-cyclase)^50^ and several genes from gibberellin biosynthesis pathways^51^. In contrast, *GmNAC085* is co-expressed with ABA-responsive genes, genes involved in aromatic amino acids metabolism, and, mainly, genes involved in protein catabolism and degradation, such as components of proteasome and endopeptidases, besides peroxidases. Interestingly, *GmNAC085* displays negative co-relation with genes controlling pigment biosynthesis and the photosynthetic apparatus assembly, including lycopene-β-cyclase, prolycopene isomerase, and the complex V of the light-harvesting system on photosystem I, indicating that these genes are expressed in divergent situations compared to *GmNAC085*.

### Ectopic expression of *GmNAC065* or *GmNAC085* promotes contrasting phenotypic and physiological changes in Arabidopsis

**Figure 4:**
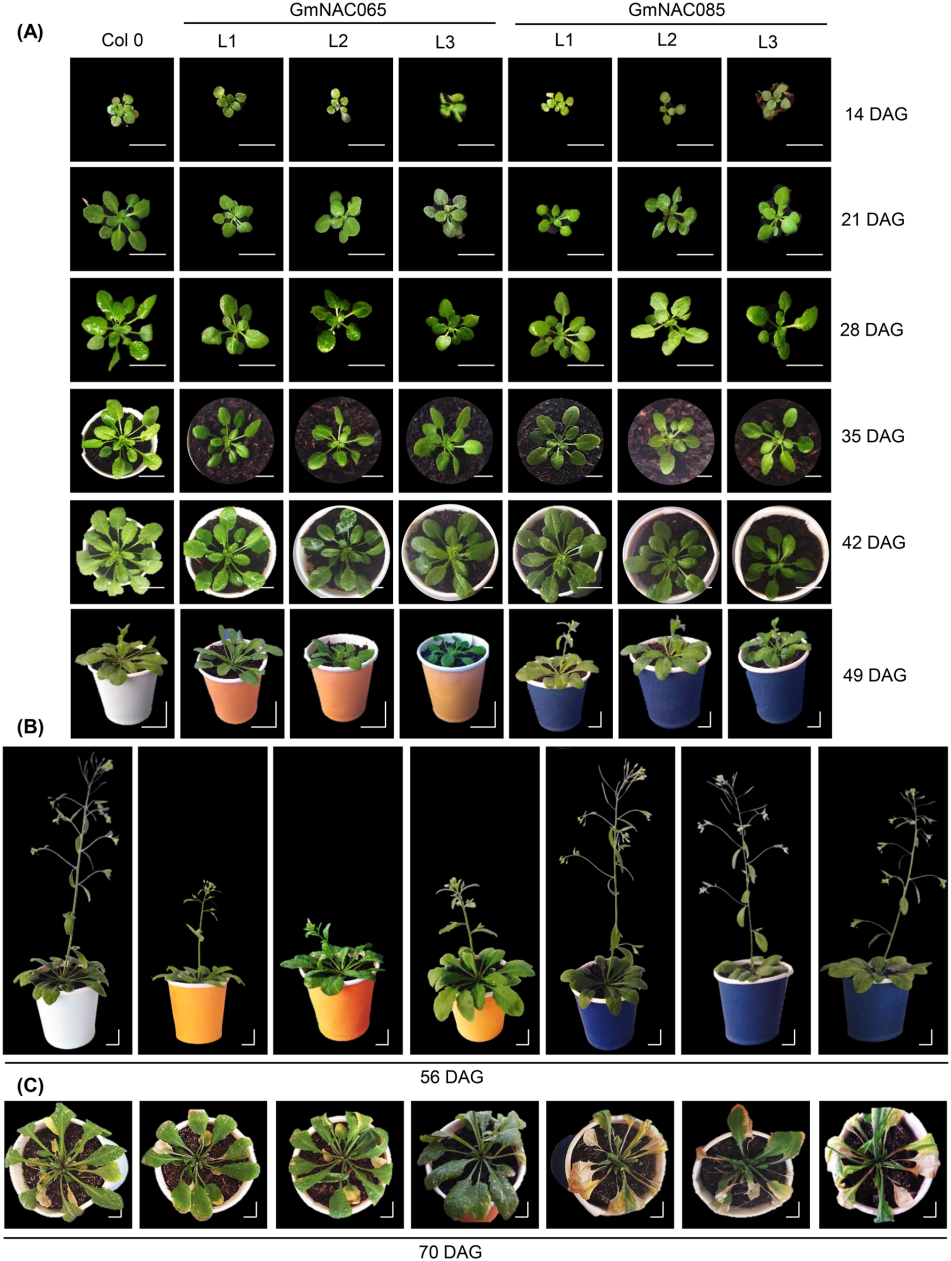
Phenotypical characterization of three homozygous independent lines of Arabidopsis overexpressing *GmNAC065* and *GmNAC085*. (**A**) Phenotypical characterization of GmNAC065-OX and GmNAC085-OX lines (L1, L2 and L3) during the vegetative stage and the onset of the reproductive stage. Plants were analyzed up to 42 days after germination (DAG) in the vegetative stage and up to 56 DAG in the reproductive stage. 49 DAG was considered the onset of the reproductive stage, hallmarked by the inflorescence’s emergence. (**B**) Phenotypical characterization of GmNAC065-OX and GmNAC085-OX lines in the reproductive stage. (**C**) Phenotypical characterization of transgenic Arabidopsis lines during senescence. All experiments were performed with plants under normal development, cultivated in a growth chamber with standard settings. Scale bars = 0.5 cm.

To uncover the functions of *GmNAC065* and *GmNAC085* in developmental and stress-responsive programs in plants, we generated three independent homozygous transgenic lines of *Arabidopsis thaliana* expressing the soybean genes under the C*aMV*35S promoter control. The selected lines (L1, L2, and L3) of GmNAC065-OX and GmNAC085-OX demonstrated similar transgenes' expression, monitored by RT-qPCR (Supplementary Figure 2A). The ectopic expression of GmNAC065 and GmNAC085 in the Columbia background caused phenotypic alterations (Fig. 4, Supplementary Figure 2). The transgenic plants displayed stunted growth compared to wild-type plants during the vegetative stage (Fig. 4A). From 14 days after germination (DAG) up to 49 DAG, considered as the transition point to the reproductive stage, hallmarked by shoot elongation in plants, the leaf number and rosette diameter of both GmNAC065-OX and GmNAC085-OX were lower compared to Col 0 (Fig. 4A, Supplementary Figure 2B,C). GmNAC065-OX plants reached an average of maximum rosette diameter of 9.88 (± 0.47) cm, against 9.80 (± 0.11) cm of GmNAC085-OX and 10.89 (± 0.94 cm) of Col 0.

During the reproductive stage, the transgenic lines also displayed lower inflorescence length than wild type plants (Fig. 4B, Supplementary Figure 2D), and the phenotypic alterations were more pronounced between the different transgenic lines. The development of GmNAC065-OX lines was delayed compared to Col 0 and GmNAC085-OX plants. At 49 DAG, Col 0 and GmNAC085-OX emerged their shoot-inflorescence, reaching 2–3 cm of inflorescence length, while only one of five GmNAC065-OX L1 plants displayed shoot elongation (Fig. 4A, Supplementary Figure 2D). At the same time point, GmNAC085-OX L1 flowered, indicating an accelerated development compared to the other plants (Fig. 4A). These phenotypic variations persisted during the late reproductive stages. At 56 and 63 DAG, Col 0 and GmNAC085-OX lines presented elongated shoots with flowers, although the inflorescence length was shorter in GmNAC085-OX than in Col 0 (Fig. 4B, Supplementary Figure 2D). The reduced number of leaves, rosette diameter, and shoot length displayed by GmNAC065-OX lines indicate a longer lifecycle and a stay-green phenotype. At 70 DAG, after flower abscission and silique maturation, GmNAC065-OX plants kept the green phenotype, with discrete senescence marks, whereas GmNAC085 OX plants displayed more intense necrotic lesions as compared to Col-0. Collectively, these phenotypic alterations indicate that the overexpression of *GmNAC065* delays the developmental cycle of transgenic plants, whereas *GmNAC085* overexpression accelerates leaf senescence, which is consistent with the GmNAC065 and GmNAC085 expression patterns in soybean under normal growth conditions and in response to multiple stresses.

### GmNAC065 and GmNAC085 ectopic expression causes changes in the antioxidant plant system and secondary metabolite content

**Figure 5:**
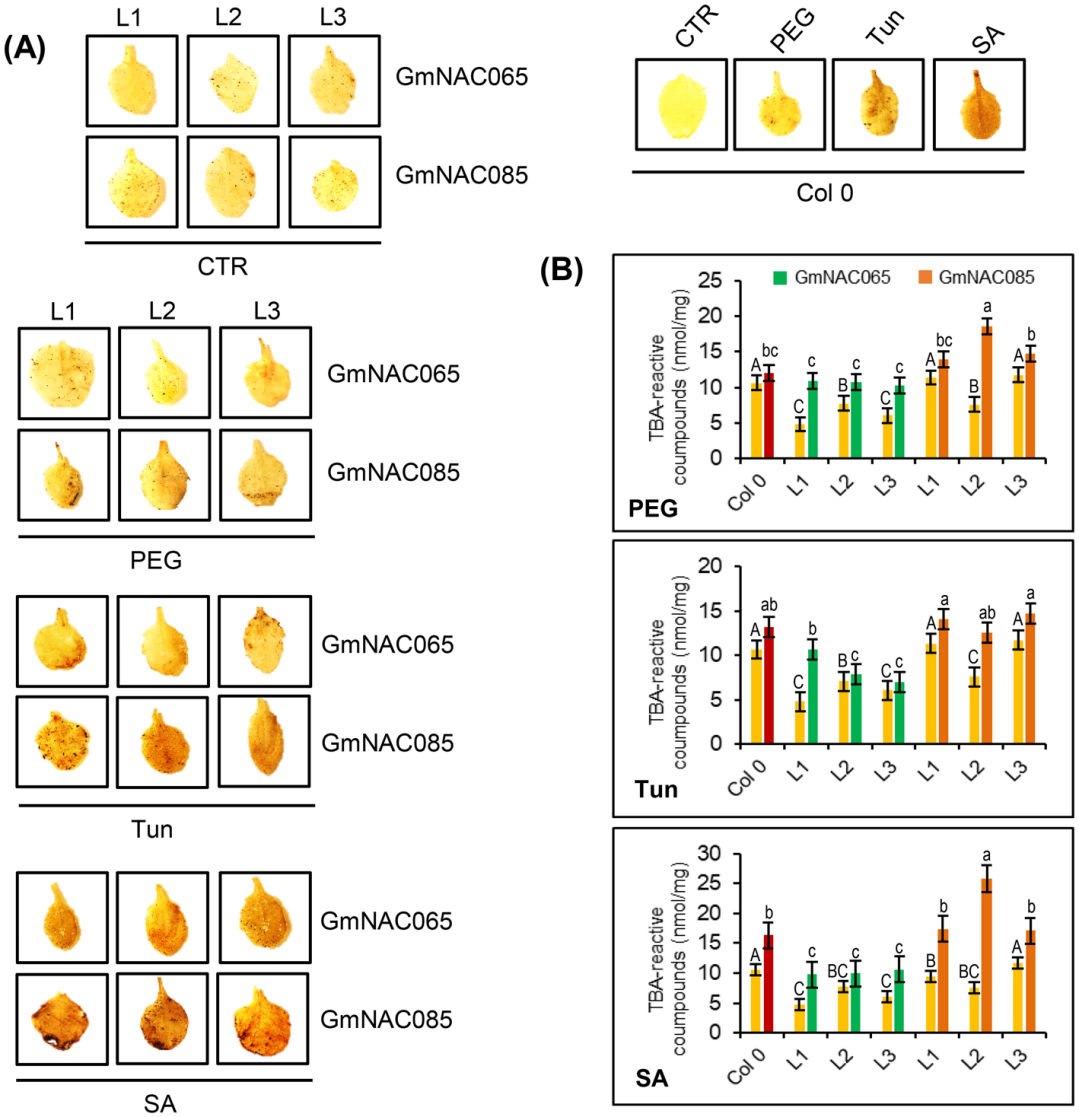
Expression of *GmNAC065* and *GmNAC085* leads to H_2_O_2_ accumulation and lipid peroxidation. (**A**) DAB-leaf staining to detect H_2_O_2_ accumulation in Arabidopsis leaves submitted to different stresses (PEG 8000—10% w/v; Tun—5 µg/mL; SA—75 µM) for 24 h. (**B**) TBA-reactive compounds quantification in GmNAC065-OX and GmNAC085-OX lines under different stresses. Solid bars show data from untreated plants. Upper bars indicate standard error (95% of confidence). Uppercase letters indicate significant differences among control samples and lowercase letters indicate significant differences among treated samples by the Tukey's test (p < 0.05, n = 3).

**Figure 6:**
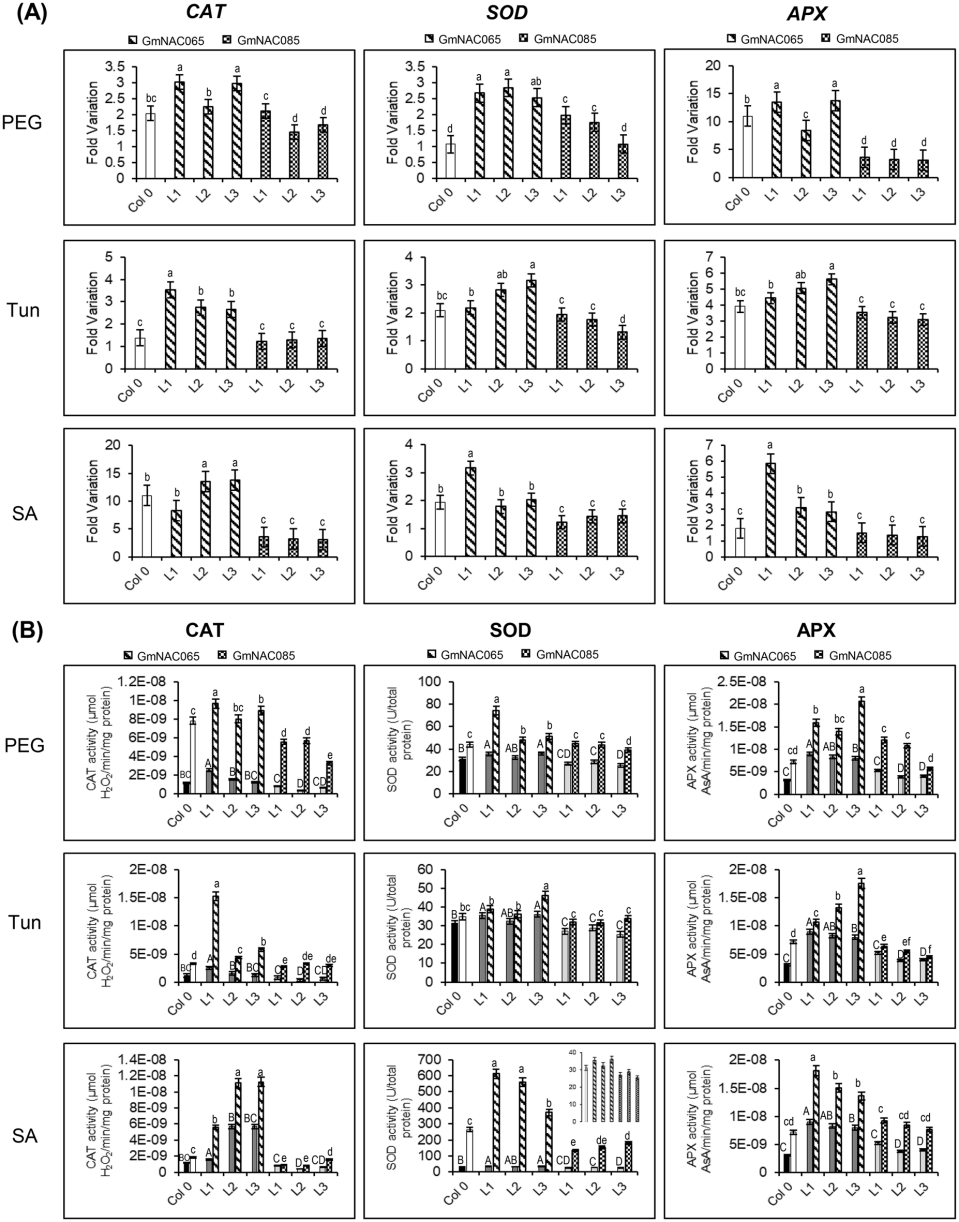
Expression profiles and activities of antioxidant enzymes catalase (CAT), superoxide dismutase (SOD), and ascorbate peroxidase (APX) in GmNAC065-OX and GmNAC085-OX lines under different stress conditions. (**A**) Expression profile of CAT, SOD, and APX in transgenic Arabidopsis lines after 24 h of stresses. Fold variation data were normalized according to the expression levels of the control samples (FV = 1.0). (**B**) Enzymatic activity of CAT, SOD, and APX in GmNAC065-OX and GmNAC085-OX plants under different stresses. Drought, ER stress, and biotic stress were simulated by PEG 8000—10% w/v; Tun—5 µg/mL; and SA—75 µM treatments, respectively. Solid bars show data from untreated plants. Upper bars indicate standard error (95% of confidence). Uppercase letters indicate significant differences among control samples, and lowercase letters indicate significant differences among treated samples by the Tukey's test (p < 0.05, n = 3).

**Figure 7:**
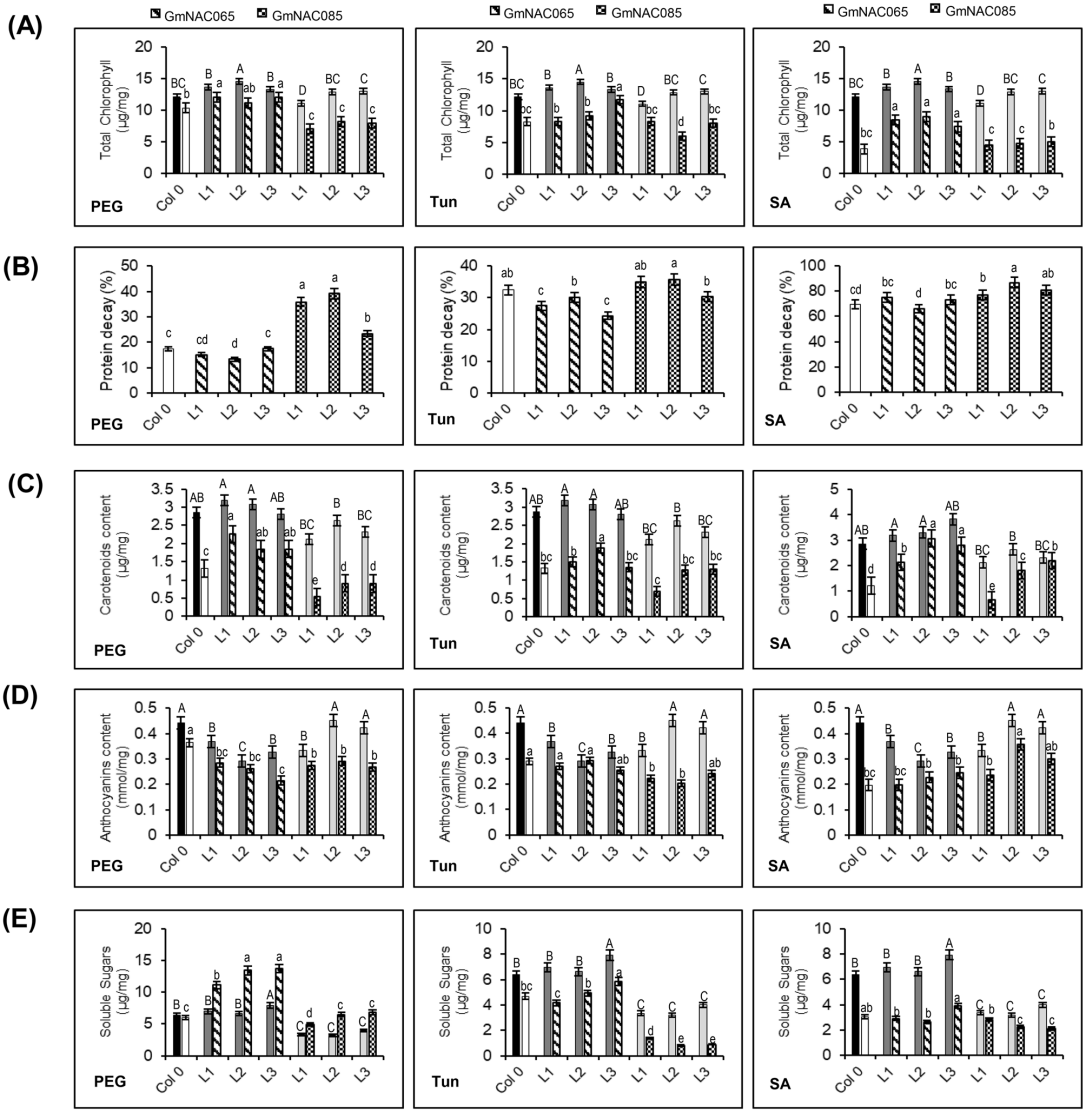
Metabolite content in transgenic Arabidopsis lines overexpressing *GmNAC065* and *GmNAC085* under different stress conditions. (**A**) Chlorophyll content. The chlorophyll content was accessed spectrophotometrically from ethanolic plant extracts. (**B**) Protein decay ratio. After stress, the protein decay ratio was calculated according to the total protein content, determined by the Bradford method. The protein content of untreated plants was considered 100% and the degradation ratio was expressed as the percentage of protein relative to the control. (**C**) Carotenoid content. The carotenoid content was determined spectrophotometrically from ethanolic plant extracts. (**D**) Anthocyanin content. Anthocyanin content was determined spectrophotometrically from ethanolic plant extracts. (**E**) Soluble sugar content was determined by the DNS-reducing method. Samples were subjected to the DNS reaction, and sugar concentration was determined based on a glucose standard curve. Drought, ER stress, and biotic stress were simulated by PEG 8000—10% w/v; Tun—5 µg/mL; and SA—75 µM treatments for 24 h, respectively. Solid bars show data from untreated plants. Upper bars indicate standard error (95% of confidence). Uppercase letters indicate significant differences among control samples, and lowercase letters indicate significant differences among the treated samples by the Tukey's test (p < 0.05, n = 3).

A common feature on plant responses to multiple stresses is the production and accumulation of reactive oxygen species (ROS)^10, 52, 53^. With the different extent of damages, they are potent oxidizers and lead to deleterious effects, triggering ePCD^10, 54^. The ectopic expression of *GmNAC065* and *GmNAC085* in *N. benthamiana* leaves has already been shown to differentially accumulate H_2_O_2_ and thiobarbituric acid (TBA)-reactive compounds, such as malonaldehyde (MDA—a product of lipid peroxidation), consequently reducing the chlorophyll content after 72 h^33^. Hereafter, we investigated the effect of ROS accumulation on activity of antioxidant enzymes and osmo- and oxidative-protectant content in Arabidopsis transgenic lines constitutively expressing these genes (Figs. 5, 6, 7).

In untreated control (CTR) plants, the levels of H_2_O_2_ were very similar, indicating that additional stress was not generated during the plant acclimation (Fig. 5A). In PEG-treated plants, GmNAC065-OX lines show a discretely lower accumulation of ROS compared to Col-0, but significantly lower ROS content compared to GmNAC085-OX plants, as demonstrated by the MDA content (Fig. 5B). Tun- and SA-treated plants display a similar pattern of TBA-reactive compounds, although the differences between GmNAC065-OX lines and GmNAC085-OX lines are accentuated. These treatments drastically impose an oxidative burst but the H_2_O_2_ and MDA contents of GmNAC065-OX plants remain lower than those of GmNAC085-OX lines, which show similar levels of these compounds as in wild type plants (Fig. 5B).

ROS accumulation emerges from imbalances in the photosynthetic apparatus and an impaired antioxidant system. In the transgenic plants, the gene induction of antioxidant enzymes catalase (CAT), superoxide-dismutase (SOD), and ascorbate-peroxidase (APX) was monitored (Fig. 6A). GmNAC065-OX lines exhibited higher induction of anti-oxidative enzymes in response to the stress inducers PEG, TUN and SA than Col-0 and GmNAC085-OX plants. The activity of antioxidant enzymes correlated with the expression levels detected in wild type and transgenic plants. GmNAC065-OX lines displayed higher enzymatic activity even in untreated plants because of the enhanced transcript accumulation of the encoded genes (Fig. 6B). Under different stresses, the activity of the anti-oxidative enzymes remains significantly higher in GmNAC065 lines, followed by Col-0 and GmNAC085-OX.

A typical symptom of ROS accumulation is the breakdown of chlorophyll and other pigments^10, 53, 55^. The total chlorophyll content of *GmNAC065-*overexpressing transgenic leaves was higher than that of Col-0 and GmNAC085-OX leaves (Fig. 7A). After 24 h of stresses, the chlorophyll content decreased, but GmNAC065-OX plants maintain the chlorophyll content higher than wild type and GmNAC085-OX plants. In contrast, GmNAC085-OX lines displayed a lower chlorophyll content in untreated plants, and exhibited a further decrease under PEG, Tun and SA treatments, similarly to wild type plants.

ROS accumulation also leads to protein and DNA degradation. The protein decay in response to stress conditions in GmNAC085-OX lines was extensively higher than in GmNAC065-OX and Col-0 plants (Fig. 7B). In response to PEG and Tun treatments, the protein content decay reached almost 40% in GmNAC085-OX lines, against 15–20% (PEG) and 25–30% (Tun) observed in GmNAC065-OX and Col 0 plants, suggesting an extensive activity of biomolecules’ breakdown system accentuated by high ROS accumulation in these plants. In SA-treated plants, the protein decay differences between the lines were attenuated, probably due to the oxidative burst caused by SA. However, GmNAC065-OX plants remained with the best performance.

The stress responses in plants involve multilayered stress-avoidance mechanisms that, along with tolerance mechanisms, confer the resistant phenotype. The co-expression analysis of genes in soybean revealed that *GmNAC065* expression positively correlates to genes involved in redox reactions, specifically those belonging to biosynthesis of pigments (Table 2). This observation prompted us to monitor other antioxidant system branches, including the carotenoids, anthocyanins and soluble sugar contents in transgenic plants (Fig. 7C–E).

The carotenoid content profile was similar to the chlorophyll pattern in the transgenic lines were similar, as *GmNAC065-*overexpressing plants exhibited higher levels of carotenoids than wild type and GmNAC085-OX plants under control and stress conditions (Fig. 7C). Curiously, anthocyanins content may not participate efficiently in the mechanisms involved in oxidative stress tolerance in GmNAC065-OX plants. Untreated GmNAC065-OX plants displayed lower anthocyanin content compared to untreated Col-0 and GmNAC085-OX plants. Under stress conditions, the anthocyanin content of GmNAC065-OX lines decreased to a similar level to Col-0, but it remained lower compared to GmNAC085-OX plants, except in response to Tun treatment (Fig. 7D).

Finally, the soluble sugars content was also analyzed. Untreated Col-0 and GmNAC065-OX lines displayed similar levels of soluble sugars in contrast to GmNAC085-OX lines, which displayed the lowest levels of these metabolites (Fig. 7E). After PEG treatment, GmNAC065-OX plants demonstrated higher sugar levels, indicating an osmoprotectant effect caused by sugar accumulation^56^. The sugar content also increased in Col 0 and GmNAC085-OX lines, but in a lower ratio compared to GmNAC065-OX plants. In other treatments, the sugar content decayed after 24 h of stress because of the extensive mobilization during ePCD triggering programs^57^ but remained higher in *GmNAC065-*expressing plants.

### *GmNAC065* and* GmNAC085* differently regulate stress-triggered programmed cell death

**Figure 8:**
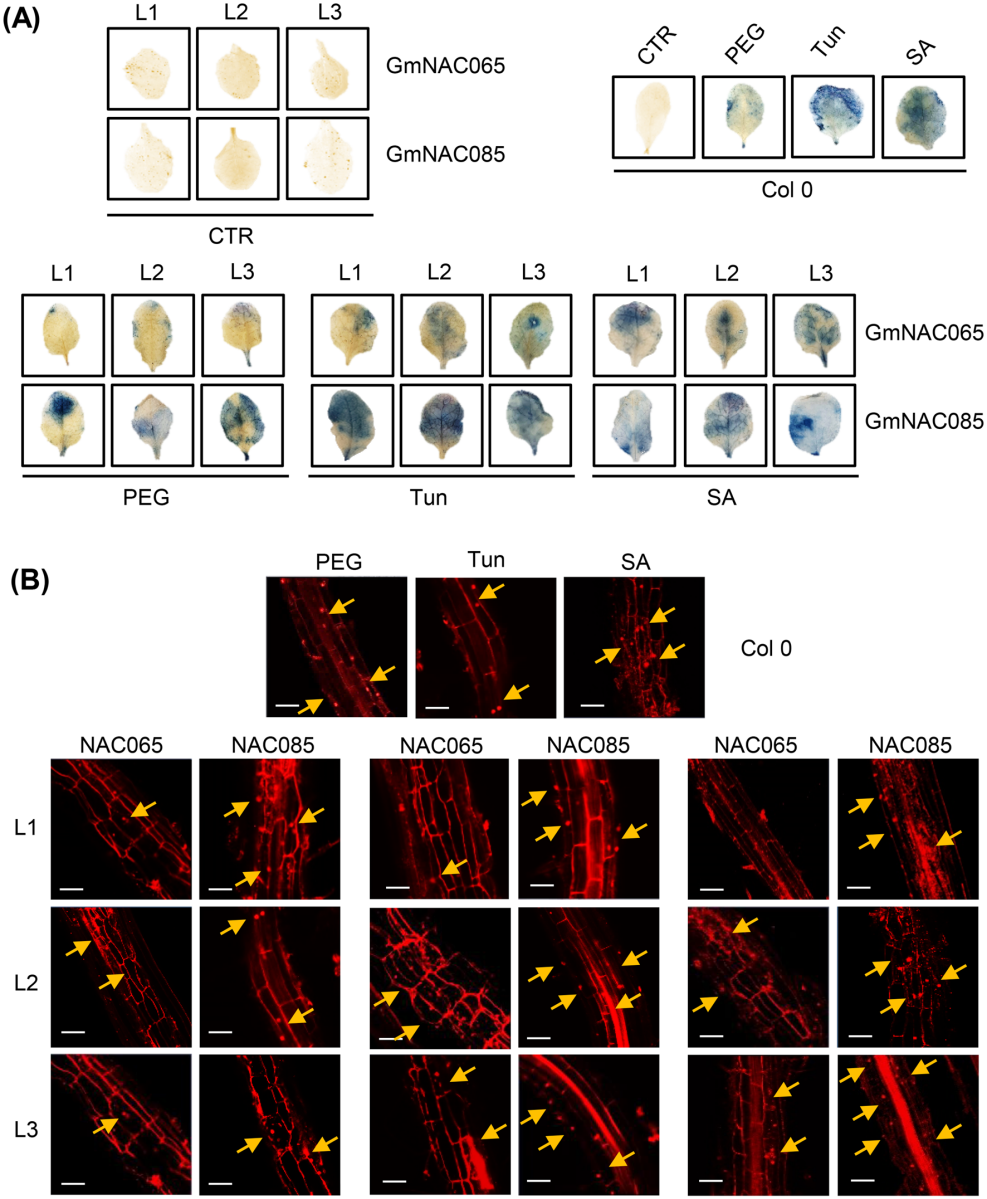
Cell death extent in leaves and roots of GmNAC065-OX and GmNAC085-OX plants. (**A**) Evans blue staining of GmNAC065-OX and GmNAC085-OX leaves under different stresses. 24 h-stressed and non-stressed leaves were exposed to the Evans dye staining solution for 8 h. The intense blue color indicates the extent of cell death. (**B**) Propidium iodide (PI) root-staining in transgenic plants ectopically expressing *GmNAC065* and *GmNAC085*. Plants were stressed with PEG 8000—10% w/v; Tun—5 µg/mL; and SA—75 µM for 24 h and the roots were subjected to PI-staining solution for 24 h until confocal microscopy scanning. PI stains the cell wall of living cells whereas dead cells show nuclei and cytoplasm stain. Yellow arrows indicate regions with extensive cell death. Scale bars = 20 µm.

**Figure 9:**
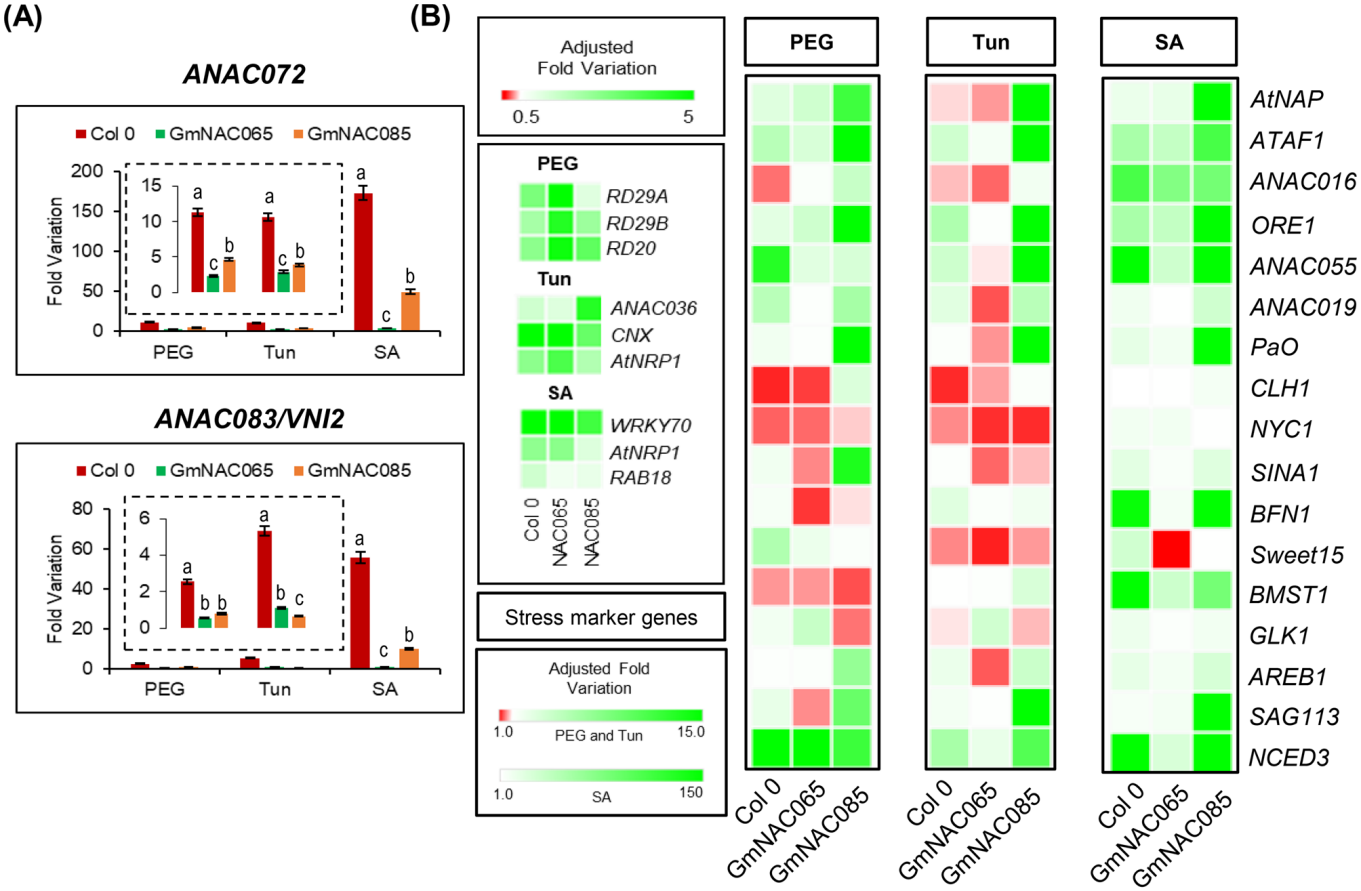
AtSAGs expression in GmNAC065-OX and GmNAC085-OX plants under different stresses. (**A**) Expression levels of *ANAC083/VNI2* and *ANAC072*, a negative and positive regulator of senescence in Arabidopsis, and the putative orthologous of *GmNAC065* and *GmNAC085*, respectively. (**B**) Expression levels of stress marker genes and AtSAGs in transgenic lines ectopically expressing *GmNAC065* and *GmNAC085*. *ACT2* was used as the endogenous control gene. Relative gene expression was quantified using the comparative 2^−ΔΔCt^ method. Upper bars indicate standard error (95% of confidence), and the different letters indicate significant differences among the lines by the Tukey's test (p < 0.05, n = 3).

**Figure 10:**
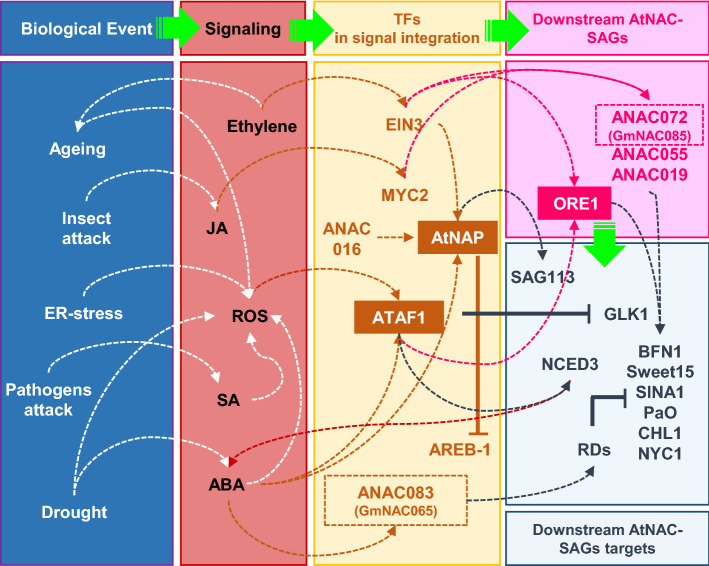
Schematic representation of the gene regulatory network coordinated by environmental stimuli, plant hormones, and AtNAC-SAGs. The different environmental stimuli culminate in plant hormone signaling, which activates several SAGs in response to multiple stresses. Consequently, the expression of these SAGs triggers an age- or -environmental cell death. *GmNAC085* up-regulates *ATAF1*, *ORE1,* and *AtNAP*, involved in integrating ABA, age, and ROS signals. Increased expression of these genes results in the activation of several downstream SAGs involved in the degradation of chlorophyll and other pigments, which confer accentuated senescence phenotype. Contrastingly, the expression of these genes is attenuated by *GmNAC065* leading to a better plant performance under multiple stresses and a delayed senescence phenotype. Adjusted from Bengoa-Luoni et al.^2^.

Our genome- and transcriptome-wide analyses, supported by the biochemical characterization of the Arabidopsis transgenic lines, have associated *GmNAC065* and *GmNAC085* with different roles in ePCD. The extent of *GmNAC065*- and *GmNC085*-mediated ePCD was monitored by Evans blue leaf-staining (Fig. 8A) and propidium iodide (PI) root-staining (Fig. 8B) after 24 h-treatment of transgenic lines with PEG, Tun and SA as well as by the transcriptional modulation of several SAGs regulated by different stimuli in Arabidopsis (Figs. 9 and 10)*.*

Evans blue is a vital dye and only penetrates dead cells, indicating the extent of cell death in leaves. Likewise, PI is a fluorescent base-intercalating dye and stains the nucleus of dead cells in roots. GmNAC065-OX lines displayed an attenuated ePCD phenotype compared to Col-0 and GmNAC085-OX, which displayed the most extensive cell death, as demonstrated by intense blue-staining in leaves and higher nuclei-staining and disrupted cells in roots (Fig. 8). These results are consistent with the phenotypic and molecular analyses of GmNAC065-OX and GmNAC085-OX lines, which converge to an accentuated ePCD-phenotype observed in GmNAC085-OX lines in contrast to the attenuated phenotype displayed by GmNAC065-OX lines.

The basis for the intensity of a senescence phenotype is the extensive gene expression reprogramming and signaling in plants^2^. In Arabidopsis, gene regulatory networks (GRNs) involved in integrating environmental and developmental signals triggering PCD are well characterized, and several NAC TFs function as regulators of these pathways^2, 44, 58, 59^. In order to clarify how *GmNAC065* and *GmNAC085* affect ePCD in Arabidopsis, the expression levels of several AtSAGs and downstream targets that promote classic PCD phenotypes were accessed in a pool of transgenic lines exposed to different stresses. We also investigated the expression of drought-associated gene-markers (*RD29A*, *RD29B*, and *RD20*), as well as ER-stress- (*ANAC036*, *CNX,* and *AtNRP1*) and SA-associated markers (*WRKY70*, *RAB18,* and *AtNRP1*).

All stress marker genes were correctly up-regulated in Col-0, GmNAC065-OX, and GmNAC085-OX plants, indicating that the stress treatments were effective (Fig. 9B). The expression levels of the putative *GmNAC065* and *GmNAC085* orthologs from Arabidopsis, the *ANAC083/VNI2* and *ANAC072 genes*, respectively, were also monitored. The induction of *ANAC083* and *ANAC072* in different stresses is not affected by the ectopic expression of the soybean orthologous genes (Fig. 9A). Nevertheless, under PEG, Tun, and SA treatments, their expression is remarkably higher in wild type plants than in transgenic lines. Interestingly, *ANAC083* in response to these stresses displays lower induction than *ANAC072*, supporting the phylogenetic analysis and the contrasting roles of these genes in ePCD, as suggested for their soybean orthologs.

In all treatments, several SAGs were up-regulated because of the severity of stress (Fig. 9B; Supplementary Table 4). In addition, many of them participate in integrative responses among environmental, hormone signaling, and adaptive responses^2^. In general, GmNAC085-OX lines display expressive induction of SAGs in all analyzed treatments, opposing GmNAC065-OX lines (Fig. 9B; Supplementary Table 4).

Overall, almost all SAGs were up-regulated in GmNAC085-OX lines and genes related to chloroplast-preserving and maintenance, including *GLK1,* was repressed in all treatments. In contrast, several SAGs and ePCD effectors were down-regulated in GmNAC065-OX (Fig. 9B; Supplementary Table 4). *AtNAP*, *ATAF1*, *ORE1*, *ANAC016*, *ANAC019* and *ANAC055* were weakly induced, and their downstream targets *NYC1* (NONYELLOW COLORING 1)*, SINA1* (SEVEN IN ABSENTIA), *BFN1* (BIFUNCTIONA NUCLEASE1), *BSMT1* (S-ADENOSYLMETHIONINE-DEPENDENT METHYL-TRANSFERASE), *GLK1* (GOLDEN2-LIKE 1)*,* and *SAG113* were down-regulated. Pivotal AtSAGs controlling downstream genes involved in chlorophyll degradation/chloroplast maintenance (including *ATAF1*, *ORE1*, *SAG113*, *AtNAP*, and *PaO*), DNA and protein degradation (*SINA1* and *BFN1*), and hormone- and ROS-mediated signaling (*ATAF1, NCED3*) were massively up-regulated in GmNAC085 by at least one tested stress.

## Discussion

TFs act as converging nodes that integrate stress signals and programmed cell death responses. NAC TFs have been frequently associated with stress and cell death responses in several plants, including tobacco, oat, rice, maize, sunflower, and soybean^4, 44, 60–66^. The comprehensive analysis of SAGs and other genes associated with the control of PCD-related mechanisms in crops offers a new insight over the cellular mechanisms of stress adaption and PCD, raising new possibilities for biotechnological intervention and development of modern agribusiness.

Wide-genome analysis of NAC superfamily in soybean identified 16 putative *Gm*NAC-SAGs, according to their phylogenetic relationship with *At*NAC-SAGs. Our analysis clustered them in 7 groups, whose members were distributed between 5 different NAC subfamilies^33^. Eight genes, corresponding to 50% of putative *Gm*NAC-SAGs, belonged to the SNAC-A and SNAC-B subfamilies. These groups harbor functionally characterized Arabidopsis NAC-SAGs that have been implicated as positive regulators of leaf senescence, including *AtNAP* (group 5), *ATAF1* (group 6), *ANAC072*, *ANAC019*, and *ANAC055* (group 7)^41, 67–69^.

The putative orthologous of *GmNAC085*, ANAC072, is implicated in the expression of chlorophyll catabolism- and nutrient management-related genes, such as *CV* (chloroplast vesiculation) and *SWEET15*, an essential gene in chloroplast protein degradation and sugar transport in Arabidopsis, respectively^42, 70^, further implicating *GmNAC085* as a positive regulator of ePCD.

Almost all NAC-SAGs are associated with the positive progression of leaf senescence. However, few NAC-SAG genes are involved in senescence delay and stress-protective mechanisms^45, 46^. The genes of groups 3 and 4 are phylogenetically divergent, and, naturally, may unroll different functions in senescence. *ANAC042/JUB1* (group 3) is involved in the control of H_2_O_2_ levels and its overexpression results not exclusively in delayed senescence, but also in enhanced plant performance under several abiotic stresses^45^. Additionally, *VNI2/ANAC083*, orthologous of *GmNAC065* (group 4), directly activates COR and RD genes, already associated with plant longevity under stressful conditions^45, 46^, as observed for *GmNAC065*.

The transcriptome-wide analysis demonstrated that many of *Gm*NAC-SAGs are differentially regulated by multiple stresses and hormones and different phylogenetic groups display different expression patterns. This divergence becomes more evident in bleomycin-treated soybean: NAM and SNAC-A and -B genes (groups 1, 2, 5, 6, and 7) are highly induced, including *GmNAC074*, *GmNAC023*, *GmNAC003*, *GmNAC109*, *GmNAC85*, and *GmNAC092*, as well as their paralogs, whereas *GmNAC106* and *GmNAC065* from groups 3 and 4 are slightly induced.

Even under normal soybean development, the contrast between *GmNAC065* and *GmNAC085* is evident. G*mNAC085* is poorly expressed in the vegetative stage and is only expressed during the reproductive stage, with accentuated levels in flowers and leaves. Since leaf senescence and abscission are linked to normal developmental programs. *GmNAC085* may be involved in developmentally programmed leaf senescence. In contrast, *GmNAC065* exhibits a stable expression in all stages, suggesting some function in plant maintenance. Under stressful conditions, *GmNAC065* and *GmNAC085* display different induction kinetics and patterns. *GmNAC065* displays an early-to-intermediate expression in response to PEG, ABA and air-dry treatments, whereas *GmNAC085* is precociously induced by these abiotic stresses and remained highly expressed during the stress period. *GmNAC043*, a GmNAC085 paralog, shows the same expression profile^35^, which reinforces their role in ePCD, triggered in advanced stages of stress-responses.

*GmNAC065* and *GmNAC085* differentially co-relate to stress-responsive genes in soybean. *GmNAC065* is co-expressed with genes involved in plant maintenance and redox homeostasis, such as LEA proteins, which are regulators of plant desiccation tolerance in normal development, mainly in seeds and under different stresses^71^*.* Furthermore, GmNAC065 shares a redox-balanced environment with enzymes involved in pigments metabolism, like the β-carotene hydroxylase 1, which converts β-carotenoids to xanthophylls^72, 73^. Likewise, acyl-CoA oxidase is activated to increase the production of reducing intermediates as a secondary product of its reaction^74^. Differently, *GmNAC085* is expressed in drastic conditions, with ROS accumulation and depletion of biomolecules. ROS play an elementary role in cell death. The oxidative burst triggers downstream processes, such as the synthesis of lignins, phytoalexins, flavonoids, phenolic compounds, and other metabolites^75–77^. Several genes related to these processes are co-expressed with *GmNAC085*, including enzymes of lignin biosynthesis, aromatic amino acids and many proteases, peroxidases and subunits of proteasome, raising a role for the gene in ePCD progress.

The ectopic expression of GmNAC065 and GmNAC085 caused growth and development retardation, decreased number of leaves and rosette diameter, and reduced inflorescence length. Since the soybean genes are associated with different signaling pathways and cell environments, it is not clear why they cause similar phenotypes in Arabidopsis. One possible explanation would be that they differentially affect growth-hormone pathways that converge to the same phenotype. *ANAC083* and *JUB1* negatively regulates brassinosteroid and gibberellin metabolism, leading to a plant dwarfism as observed in GmNAC065-OX plants^78^. Additionally, *VNI2*-OX plants (the *GmNAC065* ortholog) also exhibited retarded growth in early stages of plant development and enhanced leaf longevity as an effect of the positive regulation of COR/RD genes^46^.

GmNAC065-OX plants also exhibited a longer life cycle, with late flowering and delayed senescence, in opposition to GmNAC085-OX plants. Accelerated senescence and growth dwarfism is also observed in other SNAC-overexpressing plants, such as *OsNAC6/SNAC2* in rice^79^ and *ATAF1* in Arabidopsis^40, 41^. Not surprisingly, the expression of *ANAC072* (the *GmNAC085* ortholog) leads to ABA-hypersensitivity, causing stunted growth, shorter inflorescences, and reduced flowers' number and seed yield, besides reduced chlorophyll content, precocious leaf yellowing, and anticipated senescence^80^.

Precedents in the literature have shown that senescence is associated with increases in ABA endogenous content and imbalances in enzymatic and non-enzymatic anti-oxidants for ROS-scavenging^10, 41, 81–83^. Our results demonstrated that GmNAC085-OX lines do not exhibit a robust anti-oxidative performance associated with lower transcription and activity of CAT, SOD, and APX, soluble sugars and carotenoids levels, and a remarkable decrease in chlorophyll content. The same phenotype is also observed in transgenic plants overexpressing other SNAC members, such as ATAF1 and ATAF2^41, 84^. Along with the enzymatic system inhibition, the non-enzymatic antioxidant system also responds weekly in the GmNAC085-OX lines. The content of carotenoids and soluble sugars in GmNAC085-OX plant are significantly lower than observed in wild type plants. The impaired enzymatic and non-enzymatic anti-oxidative systems of GmNAC085-OX lines result in the highest protein decay ratio in response to multiple stresses and the activation of ePCD mechanisms, observed as extensive cell damage in leaves and roots. Only the anthocyanin content in GmNAC085-OX lines is not lower than those observed in GmNAC065-OX and Col-0 plants, suggesting a compensatory mechanism for oxidative stress tolerance once the ectopic expression of the soybean gene leads to an impaired antioxidant system.

In contrast, GmNAC065-OX lines do not accumulate high ROS levels displaying lower MDA production, lower protein decay ratio, and less-extensive cell damage in leaves and roots. These attenuated stress-induced cell death phenotypes may result from an improved antioxidant system, mainly associated with the differential regulation of age- and-stress integrated GRNs. In GmNAC085-OX lines, the different stresses tested massively up-regulated the central regulators of senescence, as *ATAF1*, *AtNAP* and *ORE1*. In Fig. 10, we summarized the effect of environmental stimuli over the plant hormones and the GRNs integrated by AtSAGs in age and stress-induced PCD, extensively reviewed by Podzimska-Sroka et al.^44^ and Bengoa-Luoni et al.^2^, and the effect of GmNAC-SAGs expression in their regulation.

The phenotypes resulting from the GmNAC085-mediated regulation of AtSAGs and their downstream targets reinforces the role of *GmNAC085* as a PCD-related gene by promoting ePCD. We can also associate these features with the up-regulation of *ATAF1*, which integrates ABA and ROS-signaling. *ATAF1* accentuates senescence symptoms by: (i) activating the expression of *ORE1*^41^; (ii) blocking *GLK1* transcription^41^; (iii) activating a feedforward regulation in ABA synthesis throughout the activation of NCED3, an essential enzyme in abscisic acid biosynthesis^85, 86^, as observed in transgenic lines overexpressing *GmNAC085*. Interestingly, the negative regulator of leaf senescence ANAC083 is also responsive to ABA^46^, as the soybean orthologous GmNAC065, further demonstrating a complex plant regulatory pathway and the intricacy of how plant hormones can trigger survival or cell death pathways.

## Conclusion

The previous characterization of stress-responsive and senescence-associated genes in soybean has associated *GmNAC065* and *GmNAC085* with different functions in the control and integration of developmentally and environmentally triggered cell death programs. The results of the genome-wide analysis performed in this investigation allowed the establishment of a strong relationship between the soybean genes and well-characterized AtNAC-SAGs. Interestingly, *GmNAC065* is a putative ortholog of *ANAC083/VNI2* and *GmNAC085* is a putative ortholog of *ANAC072*. These Arabidopsis genes belong to different SAGs groups and exhibit contrasting senescence functions, as suggested for the soybean genes. *GmNAC065* and *GmNAC085* are differentially induced by biotic and abiotic stresses, as well as by natural- and bleomycin-induced PCD in soybean. Additionally, they display different expression profiles in soybean tissues during reproductive and vegetative stages. The expression of *GmNAC085* is higher in advanced life-stages and tissues, in which the senescence process is more evident, such as leaves, stems, and flowers. Co-expression analysis in soybean revealed that *GmNAC065* is positively co-expressed with anti-oxidative-associated genes, whereas *GmNAC085* is positively co-expressed with genes involved in pigments and protein breakdown, and hormone signaling. Finally, the characterization of transgenic Arabidopsis lines ectopically expressing *GmNAC065* and *GmNAC085* offered new insights into the function of these soybean genes in dPCD and ePCD. GmNAC065-OX lines harbor a robust enzymatic and non-enzymatic antioxidant system, with higher chlorophyll, carotenoid and soluble sugar contents, besides up-regulation and increased activity of anti-oxidant enzymes. Moreover, the expression of positive-regulators of senescence in Arabidopsis is attenuated in these lines, conferring them a senescence-delayed phenotype under multiple stresses and normal development. In contrast, in the GmNAC085-OX lines, key regulators of ABA-, SA- and ROS-mediated programmed cell death are up-regulated, contributing to the accelerated senescence phenotype in the same conditions. Collectively, our data provide new insights into the integrated regulatory mechanisms of normal development, stress-responses, and programmed cell death, implicating GmNACs, especially *GmNAC065* and *GmNAC085*, as suitable targets for biotechnology intervention and modern crop breeding.

## Supplementary Information

Supplementary Figure 1.

Supplementary Figure 2.

Supplementary Table 1.

Supplementary Table 2.

Supplementary Table 3.

Supplementary Table 4.

## References

[1] Kim HJ, Nam HG, Lim PO (2016). Regulatory network of NAC transcription factors in leaf senescence. Curr. Opin. Plant Biol..

[2] BengoaLuoni S (2019). Transcription factors associated with leaf senescence in crops. Plants.

[3] Buchanan-Wollaston V (2003). The molecular analysis of leaf senescence—A genomics approach. Plant Biotechnol. J..

[4] Lim PO, Kim HJ, Nam HG (2007). Leaf senescence. Annu. Rev. Plant Biol..

[5] Olvera-Carrillo Y (2015). A conserved core of programmed cell death indicator genes discriminates developmentally and environmentally induced programmed cell death in plants. Plant Physiol..

[6] Woo HR, Kim HJ, Lim PO, Nam HG (2019). Leaf senescence: Systems and dynamics aspects. Annu. Rev. Plant Biol..

[7] Lam E (2004). Controlled cell death, plant survival and development. Nat. Rev. Mol. Cell Biol..

[8] Thompson BG, Lake BH (1987). The effect of radiation on the long term productivity of a plant based CELSS. Adv. Space Res..

[9] Leshem YY (1988). Plant senescence processes and free radicals. Free Radic. Biol. Med..

[10] Petrov V, Hille J, Mueller-Roeber B, Gechev TS (2015). ROS-mediated abiotic stress-induced programmed cell death in plants. Front. Plant Sci..

[11] Oh SA (1997). Identification of three genetic loci controlling leaf senescence in *Arabidopsis thaliana*. Plant J..

[12] Woo HR (2001). ORE9, an F-box protein that regulates leaf senescence in Arabidopsis. Plant Cell.

[13] Woo HR, Kim HJ, Nam HG, Lim PO (2013). Plant leaf senescence and death—Regulation by multiple layers of control and implications for aging in general. J. Cell Sci..

[14] Breeze E (2011). High-resolution temporal profiling of transcripts during Arabidopsis leaf senescence reveals a distinct chronology of processes and regulation. Plant Cell.

[15] Buchanan-Wollaston V (2005). Comparative transcriptome analysis reveals significant differences in gene expression and signalling pathways between developmental and dark/starvation-induced senescence in Arabidopsis. Plant J..

[16] Puranik S, Sahu PP, Srivastava PS, Prasad M (2012). NAC proteins: Regulation and role in stress tolerance. Trends Plant Sci..

[17] Nakashima K, Takasaki H, Mizoi J, Shinozaki K, Yamaguchi-Shinozaki K (2012). NAC transcription factors in plant abiotic stress responses. Biochim. Biophys. Acta.

[18] Chen X (2014). The NAC family transcription factor OsNAP confers abiotic stress response through the ABA pathway. Plant Cell Physiol..

[19] Zhou Y (2013). Identification and functional characterization of a rice NAC gene involved in the regulation of leaf senescence. BMC Plant Biol..

[20] Liang C (2014). OsNAP connects abscisic acid and leaf senescence by fine-tuning abscisic acid biosynthesis and directly targeting senescence-associated genes in rice. Proc. Natl. Acad. Sci. U.S.A..

[21] Ricachenevsky FK, Menguer PK, Sperotto RA (2013). kNACking on heaven’s door: How important are NAC transcription factors for leaf senescence and Fe/Zn remobilization to seeds?. Front. Plant Sci..

[22] Sakuraba Y, Han S-H, Lee S-H, Hörtensteiner S, Paek N-C (2016). Arabidopsis NAC016 promotes chlorophyll breakdown by directly upregulating STAYGREEN1 transcription. Plant Cell Rep..

[23] El Mannai Y, Akabane K, Hiratsu K, Satoh-Nagasawa N, Wabiko H (2017). The NAC transcription factor gene osy37 (ONAC011) promotes leaf senescence and accelerates heading time in rice. Int. J. Mol. Sci..

[24] Chung PJ, Jung H, Choi YD, Kim J-K (2018). Genome-wide analyses of direct target genes of four rice NAC-domain transcription factors involved in drought tolerance. BMC Genomics.

[25] Irsigler AST (2007). Expression profiling on soybean leaves reveals integration of ER- and osmotic-stress pathways. BMC Genomics.

[26] Mendes GC (2013). GmNAC30 and GmNAC81 integrate the endoplasmic reticulum stress- and osmotic stress-induced cell death responses through a vacuolar processing enzyme. Proc. Natl. Acad. Sci. U.S.A..

[27] Costa MDL (2008). A new branch of endoplasmic reticulum stress signaling and the osmotic signal converge on plant-specific asparagine-rich proteins to promote cell death. J. Biol. Chem..

[28] Faria JAQA (2011). The NAC domain-containing protein, GmNAC6, is a downstream component of the ER stress- and osmotic stress-induced NRP-mediated cell-death signaling pathway. BMC Plant Biol..

[29] Reis PAA (2011). The binding protein BiP attenuates stress-induced cell death in soybean via modulation of the N-rich protein-mediated signaling pathway. Plant Physiol..

[30] Hara-Nishimura I, Hatsugai N, Nakaune S, Kuroyanagi M, Nishimura M (2005). Vacuolar processing enzyme: An executor of plant cell death. Curr. Opin. Plant Biol..

[31] Reis PAB (2016). Functional and regulatory conservation of the soybean ER stress-induced DCD/NRP-mediated cell death signaling in plants. BMC Plant Biol..

[32] Pimenta MR (2016). The Stress-induced soybean NAC transcription factor GmNAC81 plays a positive role in developmentally programmed leaf senescence. Plant Cell Physiol..

[33] Melo BP (2018). Revisiting the soybean gmnac superfamily. Front. Plant Sci..

[34] Le DT (2011). Genome-wide survey and expression analysis of the plant-specific NAC transcription factor family in soybean during development and dehydration stress. DNA Res..

[35] Freitas EO (2019). Identification and characterization of the GmRD26 soybean promoter in response to abiotic stresses: Potential tool for biotechnological application. BMC Biotechnol..

[36] Clough SJ, Bent AF (1998). Floral dip: A simplified method for *Agrobacterium*-mediated transformation of *Arabidopsis thaliana*. Plant J..

[37] de Melo BP (2020). Transcriptional modulation of AREB-1 by CRISPRa improves plant physiological performance under severe water deficit. Sci. Rep..

[38] Sims DA, Gamon JA (2002). Relationships between leaf pigment content and spectral reflectance across a wide range of species, leaf structures and developmental stages. Remote Sens. Environ..

[39] Tran L-SP (2004). Isolation and functional analysis of Arabidopsis stress-inducible NAC transcription factors that bind to a drought-responsive cis-element in the early responsive to dehydration stress 1 promoter. Plant Cell.

[40] Wu Y (2009). Dual function of Arabidopsis ATAF1 in abiotic and biotic stress responses. Cell Res..

[41] Garapati P, Xue G-P, Munné-Bosch S, Balazadeh S (2015). Transcription factor ATAF1 in arabidopsis promotes senescence by direct regulation of key chloroplast maintenance and senescence transcriptional cascades. Plant Physiol..

[42] Li X (2016). Dual function of NAC072 in ABF3-mediated ABA-responsive gene regulation in Arabidopsis. Front. Plant Sci..

[43] Carvalho HH (2014). The molecular chaperone binding protein BiP prevents leaf dehydration-induced cellular homeostasis disruption. PLoS One.

[44] Podzimska-Sroka D, O’Shea C, Gregersen PL, Skriver K (2015). NAC transcription factors in senescence: From molecular structure to function in crops. Plants.

[45] Wu A (2012). JUNGBRUNNEN1, a reactive oxygen species-responsive NAC transcription factor, regulates longevity in Arabidopsis. Plant Cell.

[46] Yang S-D, Seo PJ, Yoon H-K, Park C-M (2011). The Arabidopsis NAC transcription factor VNI2 integrates abscisic acid signals into leaf senescence via the COR/RD genes. Plant Cell.

[47] Davison PA, Hunter CN, Horton P (2002). Overexpression of beta-carotene hydroxylase enhances stress tolerance in Arabidopsis. Nature.

[48] Saha B, Borovskii G, Panda SK (2016). Alternative oxidase and plant stress tolerance. Plant Signal. Behav..

[49] Magwanga RO (2018). Characterization of the late embryogenesis abundant (LEA) proteins family and their role in drought stress tolerance in upland cotton. BMC Genet..

[50] Stenzel I (2003). Jasmonate biosynthesis and the allene oxide cyclase family of *Arabidopsis thaliana*. Plant Mol. Biol..

[51] Yamaguchi S (2008). Gibberellin metabolism and its regulation. Annu. Rev. Plant Biol..

[52] Jaspers P, Kangasjärvi J (2010). Reactive oxygen species in abiotic stress signaling. Physiol. Plant..

[53] Petrov VD, Van Breusegem F (2012). Hydrogen peroxide-a central hub for information flow in plant cells. AoB Plants.

[54] Gechev TS, Hille J (2005). Hydrogen peroxide as a signal controlling plant programmed cell death. J. Cell Biol..

[55] Torres MA, Jones JDG, Dangl JL (2006). Reactive oxygen species signaling in response to pathogens. Plant Physiol..

[56] Puniran-Hartley N, Hartley J, Shabala L, Shabala S (2014). Salinity-induced accumulation of organic osmolytes in barley and wheat leaves correlates with increased oxidative stress tolerance: In planta evidence for cross-tolerance. Plant Physiol. Biochem..

[57] Shi H, Wang B, Yang P, Li Y, Miao F (2016). Differences in sugar accumulation and mobilization between sequential and non-sequential senescence wheat cultivars under natural and drought conditions. PLoS One.

[58] Kim HJ (2014). Gene regulatory cascade of senescence-associated NAC transcription factors activated by ETHYLENE-INSENSITIVE2-mediated leaf senescence signalling in Arabidopsis. J. Exp. Bot..

[59] Jensen MK, Skriver K (2014). NAC transcription factor gene regulatory and protein-protein interaction networks in plant stress responses and senescence. IUBMB Life.

[60] Even-Chen Z, Itai C (1975). The role of abscisic acid in senescence of detached tobacco leaves. Physiol. Plant..

[61] Gepstein S, Thimann KV (1980). Changes in the abscisic acid content of oat leaves during senescence. Proc. Natl. Acad. Sci. U.S.A..

[62] Philosoph-Hadas S, Hadas E, Aharoni N (1993). Characterization and use in ELISA of a new monoclonal antibody for quantitation of abscisic acid in senescing rice leaves. Plant Growth Regul..

[63] Quirino BF, Noh YS, Himelblau E, Amasino RM (2000). Molecular aspects of leaf senescence. Trends Plant Sci..

[64] Olsen AN, Ernst HA, Leggio LL, Skriver K (2005). NAC transcription factors: Structurally distinct, functionally diverse. Trends Plant Sci..

[65] He P, Osaki M, Takebe M, Shinano T, Wasaki J (2005). Endogenous hormones and expression of senescence-related genes in different senescent types of maize. J. Exp. Bot..

[66] de Camargos LF (2018). Development and cell death domain-containing asparagine-rich protein (DCD/NRP): An essential protein in plant development and stress responses. Theor. Exp. Plant Physiol..

[67] Guo Y, Gan S (2006). AtNAP, a NAC family transcription factor, has an important role in leaf senescence. Plant J..

[68] Rauf M (2013). NAC transcription factor speedy hyponastic growth regulates flooding-induced leaf movement in Arabidopsis. Plant Cell.

[69] Hickman R (2013). A local regulatory network around three NAC transcription factors in stress responses and senescence in Arabidopsis leaves. Plant J..

[70] Kamranfar I (2018). Transcription factor RD26 is a key regulator of metabolic reprogramming during dark-induced senescence. New Phytol..

[71] Dalal M, Tayal D, Chinnusamy V, Bansal KC (2009). Abiotic stress and ABA-inducible Group 4 LEA from *Brassica napus* plays a key role in salt and drought tolerance. J. Biotechnol..

[72] Kim JH (2009). Trifurcate feed-forward regulation of age-dependent cell death involving miR164 in Arabidopsis. Science.

[73] Arango J, Jourdan M, Geoffriau E, Beyer P, Welsch R (2014). Carotene hydroxylase activity determines the levels of both α-carotene and total carotenoids in orange carrots. Plant Cell.

[74] Arent S, Pye VE, Henriksen A (2008). Structure and function of plant acyl-CoA oxidases. Plant Physiol. Biochem..

[75] Lamb CJ, Lawton MA, Dron M, Dixon RA (1989). Signals and transduction mechanisms for activation of plant defenses against microbial attack. Cell.

[76] Cutt JR, Klessig DF, Boller T, Meins F (1992). Genes Involved in Plant Defense.

[77] Leon J, Lawton MA, Raskin I (1995). Hydrogen peroxide stimulates salicylic acid biosynthesis in tobacco. Plant Physiol..

[78] Shahnejat-Bushehri S, Tarkowska D, Sakuraba Y, Balazadeh S (2016). Arabidopsis NAC transcription factor JUB1 regulates GA/BR metabolism and signalling. Nat. Plants.

[79] Nakashima K (2007). Functional analysis of a NAC-type transcription factor OsNAC6 involved in abiotic and biotic stress-responsive gene expression in rice. Plant J..

[80] Fujita M (2004). A dehydration-induced NAC protein, RD26, is involved in a novel ABA-dependent stress-signaling pathway. Plant J..

[81] Lee IC (2011). Age-dependent action of an ABA-inducible receptor kinase, RPK1, as a positive regulator of senescence in Arabidopsis leaves. Plant Cell Physiol..

[82] Finkelstein R (2013). Abscisic Acid synthesis and response. Arabidopsis Book.

[83] Zhu J-K (2016). Abiotic stress signaling and responses in plants. Cell.

[84] Nagahage ISP (2020). An Arabidopsis NAC domain transcription factor, ATAF2, promotes age-dependent and dark-induced leaf senescence. Physiol. Plant..

[85] Iuchi S (2001). Regulation of drought tolerance by gene manipulation of 9-cis-epoxycarotenoid dioxygenase, a key enzyme in abscisic acid biosynthesis in Arabidopsis. Plant J..

[86] Jensen MK (2013). ATAF1 transcription factor directly regulates abscisic acid biosynthetic gene NCED3 in Arabidopsis thaliana. FEBS Open Bio.

