# Supplementary material for: Contrasting roles of GmNAC065 and GmNAC085 in natural senescence, plant development, multiple stresses and cell death responses
Source: Sci Rep. 2021 May 27;11:11178. doi: 10.1038/s41598-021-90767-6 (PMC8160357; doi:10.1038/s41598-021-90767-6)
Supplement: Supplementary file 1 — Supplementary Figure 1. [file 41598_2021_90767_MOESM1_ESM.docx]

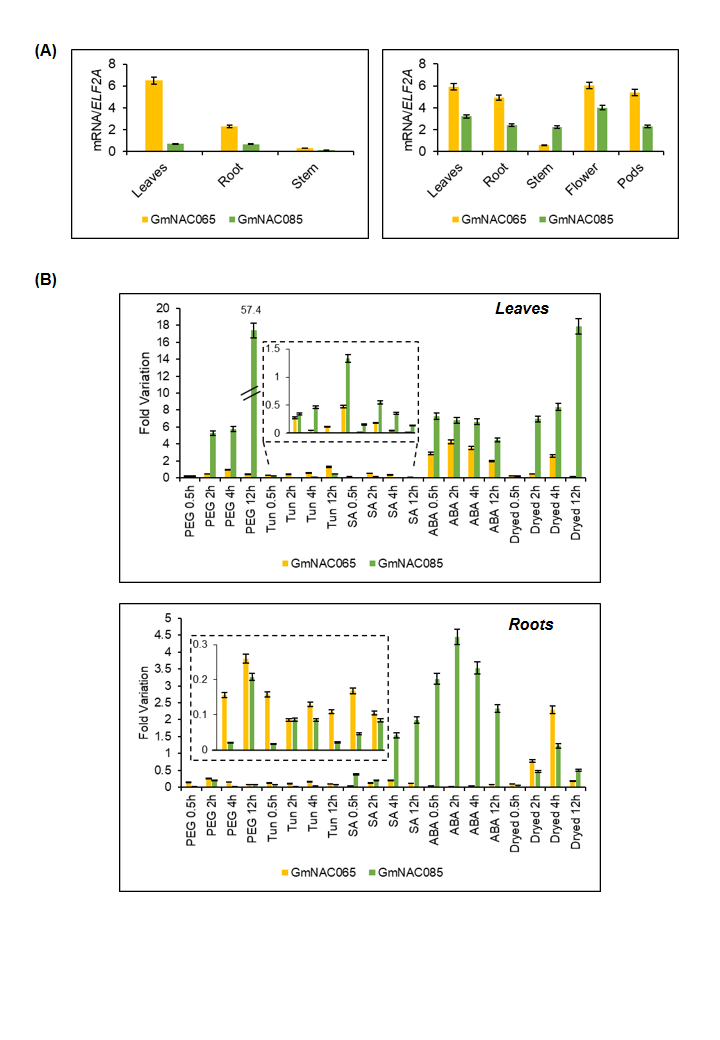


**Supplementary Figure 1. (A)** Expression levels of *GmNAC065* and *GmNAC085* in different soybean Williams82 tissues in vegetative (left) and reproductive (right) stages. Gene expression was monitored by RT-qPCR and expressed in a relative way, using *ELF1A* as the endogenous normalizer-control gene*.* **(B)** Expression levels of soybean *GmNAC065* and *GmNAC085* in roots and leaves submitted to different stresses. Transcript accumulation of the indicated genes was measured by RT-qPCR. *ELF1A* was used as the normalizer, endogenous control gene. Fold variation was calculated using the comparative 2^-ΔΔCt^ method. Analyses were performed in three biological and two technical replicates supported by Z-score normalization. Bars indicate standard error.
