# Supplementary material for: Contrasting roles of GmNAC065 and GmNAC085 in natural senescence, plant development, multiple stresses and cell death responses
Source: Sci Rep. 2021 May 27;11:11178. doi: 10.1038/s41598-021-90767-6 (PMC8160357; doi:10.1038/s41598-021-90767-6)
Supplement: Supplementary file 2 — Supplementary Figure 2. [file 41598_2021_90767_MOESM2_ESM.docx]

**
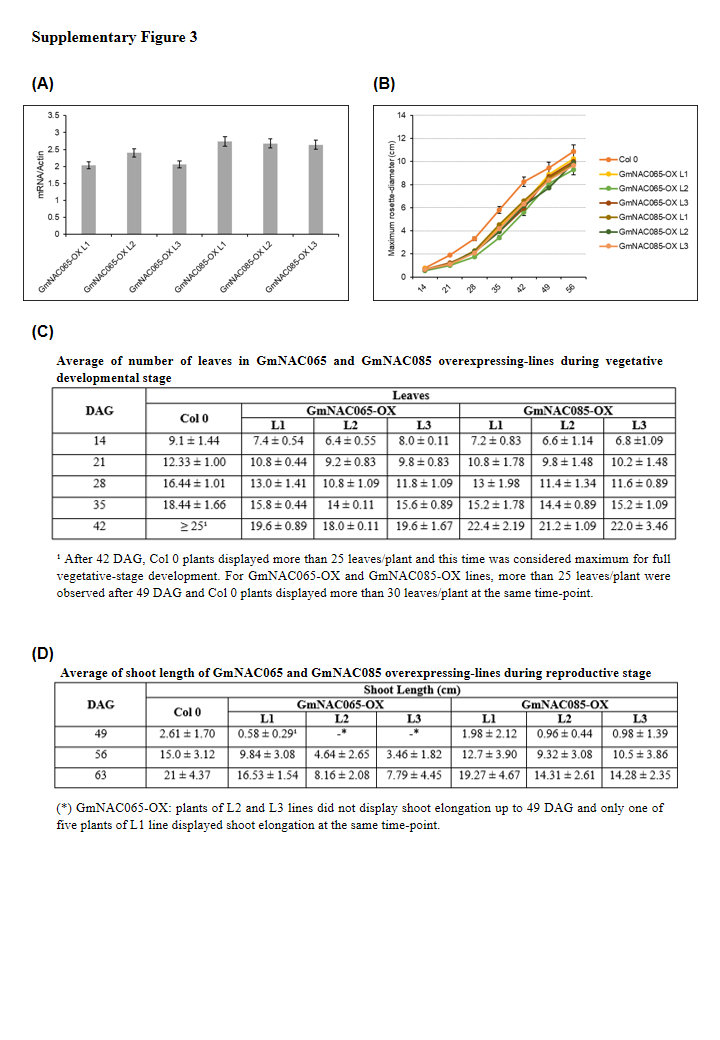
**

**Supplementary Figure 2.** Phenotypical and molecular characterization of GmNAC065-OX and GmNAC085-OX lines (L1, L2 and L3). **(A)**Transcript accumulation of the transgene *GmNAC065* and *GmNAC085* in transgenic Arabidopsis lines. Total RNA was isolated from 14 DAG T2-homozygous lines and transcript accumulation determined as the relative expression of the soybean gene and *ACT2* endogenous control, calculated by 2^-ΔCt^ method. **(B)** Rosette diameter of transgenic lines during the vegetative stage and the onset of reproductive stages. **(C)** Number of leaves data of GmNAC065 and GmNAC085-OX lines. **(D)** Rosette diameter data of GmNAC065 and GmNAC085-OX lines.
