# Supplementary material for: Contrasting roles of GmNAC065 and GmNAC085 in natural senescence, plant development, multiple stresses and cell death responses
Source: Sci Rep. 2021 May 27;11:11178. doi: 10.1038/s41598-021-90767-6 (PMC8160357; doi:10.1038/s41598-021-90767-6)
Supplement: Supplementary file 3 — Supplementary Table 1. [file 41598_2021_90767_MOESM3_ESM.docx]

**Supplementary Table 1**. Raw datasets available in GEO – NCBI used in putative GmSAGs expression-analysis in soybean under multiple stresses and senescence.

| Stress | Treatment/Time | NCBI-access number | Reference |
| --- | --- | --- | --- |
| Moderate Drought | Water withdraw – 7 days | PRJNA324522 | Chen *et al*., 2016 |
| Severe Drought | Water withdraw – 25 days | GSE50408 | Carvalho *et al*., 2014 |
| Oxidative Stress | Plant intermittent-flooding – 7 days | PRJNA324522 | Chen *et al*., 2016 |
| *Fusarium oxysporum* infection | 1.10^16^ conidial aspersion - 72 hours | GSE66861 | Lanubile *et al*., 2015 |
| *Sclerotinia sclerotiorum* infection | Simulated infection by oxalic acid (5.0 mM) vacuum-infiltration – 2 hours | GSE15369 | Calla *et al*., 2014 |
| *Lamprosema indicate* attack | Insect artificial attack | SRA549 | Zeng *et al*., 2017 |
| Ethylene-induced senescence | Leaves-explants under 25 µM of ethylene pressure – 12 hours | SRP050050 | Kim *et al*., 2018 |
| Age-induced senescence | Late R7 developmental stage leaves | GSE122915 | Melo *et al*., 2018 |

**REFERENCES**

Calla, B., Blahut-Beatty, L., Koziol, L., Simmonds, D. H., and Clough, S. J. (2014). Transcriptome analyses suggest a disturbance of iron homeostasis in soybean leaves during white mould disease establishment. *Mol. Plant Pathol.* 15, 576–588. doi:10.1111/mpp.12113.

Carvalho, H. H., Brustolini, O. J. B., Pimenta, M. R., Mendes, G. C., Gouveia, B. C., Silva, P. A., Silva, J. C. F., Mota, C. S., Soares-Ramos, J. R. L., and Fontes, E. P. B. (2014). The molecular chaperone binding protein BiP prevents leaf dehydration-induced cellular homeostasis disruption. *PLoS ONE* 9, e86661. doi:10.1371/journal.pone.0086661.

Chen, W., Yao, Q., Patil, G. B., Agarwal, G., Deshmukh, R. K., Lin, L., Wang, B., Wang, Y., Prince, S. J., Song, L., et al. (2016). Identification and Comparative Analysis of Differential Gene Expression in Soybean Leaf Tissue under Drought and Flooding Stress Revealed by RNA-Seq. *Front. Plant Sci.* 7, 1044. doi:10.3389/fpls.2016.01044.

Kim, J., Yang, J., Yang, R., Sicher, R. C., Chang, C., and Tucker, M. L. (2016). Transcriptome analysis of soybean leaf abscission identifies transcriptional regulators of organ polarity and cell fate. *Front. Plant Sci.* 7, 125. doi:10.3389/fpls.2016.00125.

Lanubile, A., Muppirala, U. K., Severin, A. J., Marocco, A., and Munkvold, G. P. (2015). Transcriptome profiling of soybean (Glycine max) roots challenged with pathogenic and non-pathogenic isolates of Fusarium oxysporum. *BMC Genomics* 16, 1089. doi:10.1186/s12864-015-2318-2.

Melo, B. P., Fraga, O. T., Silva, J. C. F., Ferreira, D. O., Brustolini, O. J. B., Carpinetti, P. A., Machado, J. P. B., Reis, P. A. B., and Fontes, E. P. B. (2018). Revisiting the soybean gmnac superfamily. *Front. Plant Sci.* 9, 1864. doi:10.3389/fpls.2018.01864.

Zeng, W., Sun, Z., Cai, Z., Chen, H., Lai, Z., Yang, S., and Tang, X. (2017). Comparative transcriptome analysis of soybean response to bean pyralid larvae. *BMC Genomics* 18, 871. doi:10.1186/s12864-017-4256-7.
