# Supplementary material for: Contrasting roles of GmNAC065 and GmNAC085 in natural senescence, plant development, multiple stresses and cell death responses
Source: Sci Rep. 2021 May 27;11:11178. doi: 10.1038/s41598-021-90767-6 (PMC8160357; doi:10.1038/s41598-021-90767-6)
Supplement: Supplementary file 4 — Supplementary Table 2. [file 41598_2021_90767_MOESM4_ESM.docx]

**Supplementary Table 2.** qRT-PCR primers used to determine the expression of *GmNAC065* and *GmNAC085* in soybean tissues and under different stresses and the expression of GmNAC-SAGs in bleomycin-treated soybean

**Endogenous Control**

**qRT_ELF1A_fwd** GACCTTCTTCGTTTCTCGCA

**qRT_ELF1A_revs** CGAACCTCTCAATCACACGC

**Target Genes**

**qRT_GmNAC065_fwd** TGGGATTTGCCAGGTGATTT

**qRT_GmNAC065_revs** GAGCGATTTCCGTTGGGATA

**qRT_GmNAC085_fwd** GCAATGGGTCATCACCTTCT

**qRT_GmNAC085_revs** GACCCAAATTCGGAAACTGA

**GmNAC-SAGs**

**qRT_GmNAC074_fwd** TCCTAGTGGTGAGCGGACTGA

**qRT_GmNAC074_revs** TAACACCCAGGCATCTCTTAAGC

**qRT_GmNAC081_fwd** GGAGCAAAGGGCAGCACTAG

**qRT_GmNAC081_revs** TGTCCGTGGAGGAAGGAGAA

**qRT_GmNAC023_fwd** CGAGCACCCAAAGGAGAGAA

**qRT_GmNAC023_revs** CGAGCACCCAAAGGAGAGAA

**qRT_GmNAC131_fwd** CTGCCACCTCTCTCGGATTC

**qRT_GmNAC131_revs** GAGAAGCAGGGCACGTAAGC

**qRT_GmNAC106_fwd** GTGTGGACGCTATGCCGAAT

**qRT_GmNAC106_revs** GGTGCTGCCGAGTCTTTCAA

**qRT_GmNAC010_fwd** AGTCCACGAGAGAGGAAGTATCCA

**qRT_GmNAC010_revs** GTGCCAGTGGCCTTCCAATA

**qRT_GmNAC109_fwd** CAACATATCGCGGTTCCCATA

**qRT_GmNAC109_revs** TACAAAGCCATTCCTGGAAGGT

**qRT_GmNAC030_fwd** GATTCCACCCCACTGACGAT

**qRT_GmNAC030_revs** GATGGGAACAGCAATGGTTTG

**qRT_GmNAC101_fwd** TCAGCCCCAGAGACAGGAAAT

**qRT_GmNAC101_revs** TCCGGTGGCTTTCCAATAAC

**qRT_GmNAC092_fwd** CGATGCCACGTGTCAACAC

**qRT_GmNAC092_revs** TAACCCCCCTTCACCCAAGT

**qRT_GmNAC149_fwd** CGGGAGATAGGCAATGGTTCT

**qRT_GmNAC149_revs** TCTGGTTGCTCGGTTAGACCTT

**qRT_GmNAC077_fwd** GGAGCAAAGGGCAGCACTAG

**qRT_GmNAC077_revs** TGGAGGAAGGAGGAGAAGCA

**qRT_GmNAC154_fwd** TCTGACAACGGCAAGCCATA

**qRT_GmNAC154_revs** GCCGGTTTCCTTTCATTTTG

**qRT_GmNAC179_fwd** TGGGATTTGCCAGGTGATTT

**qRT_GmNAC179_revs** GAGCGATTTCCGTTGGGATA

**qRT_GmNAC003_fwd** TGTACTCCCACCTGGCTTCAG

**qRT_GmNAC003_revs** TTGATGAGGCTTGGTTGCAA

**qRT_GmNAC011_fwd** TACCCCCTGGATTCCGATTC

**qRT_GmNAC011_revs** TTCTTGCGAAGCGCATTTG

**qRT_GmNAC043_fwd** GGATCGTTGCCGGAGATAAA

**qRT_GmNAC043_revs** TCATCCTGCTGGTGCATTGT
