# Supplementary material for: Contrasting roles of GmNAC065 and GmNAC085 in natural senescence, plant development, multiple stresses and cell death responses
Source: Sci Rep. 2021 May 27;11:11178. doi: 10.1038/s41598-021-90767-6 (PMC8160357; doi:10.1038/s41598-021-90767-6)
Supplement: Supplementary file 5 — Supplementary Table 3. [file 41598_2021_90767_MOESM5_ESM.docx]

**Supplementary Table 3.** qRT-PCR primers used to determine the expression of anti-oxidant enzymes, stress marker genes and AtNAC-SAGs in *Arabidopsis thaliana*

**Endogenous Control**

**ACT2-F** GATCTCCAAGGCCGAGTATGAT

**ACT2-R** CCCATTCATAAAACCCCAGC

**Anti-oxidant enzymes CAT, SOD and APX**

**APX-F** CCTCCGGAGGGTATCGTTATCTA

**APX-R** ACAGCCAGAAACATTGTCCAAAAGG

**CAT1-F** TGGGATTCAGACAGGCAAGAACG

**CAT1-R** GTTTGGCCTCACGTTAAGACGAGT

**CSD1-F** TGAACTCAGCCTGGCTACTGG

**CSD1-R** AGCCACACACCAGAAGATACACAC

**Stress marker genes**

Drought

**RD29A-F** GATTTCTTCTGATCGACAAAACCTA

**RD29A-R** AGCAAACCCAACTTATTACATTACG

**RD29B-F** GCAAGCAGAAGAACCAATCA

**RD29B-R** CTTTGGATGCTCCCTTCTCA

**RD20-F** TTAGCTCCGGTCACCAGTCA

**RD20-R** CATGTATGGTTTTGGTAATGTTTCC

ER-stress

**ANAC036 – F**  GCTCAAGAAGACGCTTGTGTT

**ANAC036 - R** CTCGTTCATCACCCAATCAG

**CNX fwd** TGATGGGGAGGAGAAGAAAAAGGC

**CNX rvs** CGGTGTAGACATGGGAAAGC

Biotic Stress

**NPR1 – F** CTGCAGACTCATACACTCTGG

**NPR1 – R** ATCCGAGTCTCACTGACTTTC

**RAB18-F** GGCTTGGGAGGAATGCTT

**RAB18-R** TTGATCTTTTGTGTTATTCCCTTCT

**AtNAC-SAGs**

**ANAC072** **- F** GCACGAGTATCGCTTAATAGAACA

**ANAC072 - R** CGACACAACACCCAATCATC

**ANAC083 – F** TTTGCAGAGCTGATCCTTGG

**ANAC083 – R** CGGTTCCCATTTGGGTATTT

**ANAC019 - F** AACTGTGGCTACCTGAAGACG

**ANAC019 - R** CCGAGTTATTAAACCCGTGACT

**AtNAP - F** GAAACCAGACCATGTCTAAACCA

**AtNAP - R** TTTCTCCAAACTCTGTTTTCTCG

**ANAC016 - F** ATTCACTTCACAGTCAACAGGTG

**ANAC016 - R** GCTGATGAGAACTGGCTCCT

**ANAC055 - F** TTCTCGAGTCGTTGCATGAG

**ANAC055 - R** CTATGAGGCAGCGCGTTT

**ORE1 - F** GTGGGTATGAAGAAAACTTTGG

**ORE1 - R** TTCGTTCTTAGCTGTTTGGGG

**ATAF1 – F** AGGCTGGATGATTGGGTTCTCTG

**ATAF1 – R** GATTTCGTCGCCGTAAACAACCG

**SAG113 - F** AGGAAAACTCAACATCCTCGTC

**SAG113 - R** GCTGACTCGAGATTTGTAGCC

**GLK1 – F** GCTACGAGATTTAGAGCACCG

**GLK1 – R** TTGACGGATGTAAGTCTACCG

**SINA1 - F** TCCTGCGAAATGGAACCTCGAATC

**SINA1 – R** TTCGATGTGGTCGTTGGGACACTC

**BFN1 – F** TTGAAGAGAAGAGTGTGGCTTGG

**BFN1 – R** AGAAGAGCCGCTTGGTCGTATG

**NCED3 – F** ACTCATGCTATT CTACGCCAGAG

**NCED3 – R** ACCAACGGTTT TTAAATCTCCAT

**Sweet15 – F** CAATGACATATGCATAGCGATTCCAA

**Sweet15 – R** GGACTCATCACGACAATACTCTTAAG

**NYC1- F** GCAGAGAACAGGACGAGGTT

**NYC1 - R** CGCAAACAACAGAAAGAGAGAA

**PaO - F** CCCAGGCAGACCGTTTTGT

**PaO - R** TGACTCTTACCATGCCGTCTGA

**CLH1 - F** CCCGTCGTTTTATTCTTCCA

**CLH 1- R** AGCATCGTCCACTTCCACTT

**BSMT1 – F** CATTCAACATGCCGTTTTATG

**BMST1 – R** CATTGGTTCACTAACAGCTC
